# The effect of renin–angiotensin–aldosterone system inhibitors on continuous and binary kidney outcomes in subgroups of patients with diabetes: a meta-analysis of randomized clinical trials

**DOI:** 10.1186/s12882-022-02763-1

**Published:** 2022-04-28

**Authors:** Noor Alsalemi, Cheryl A. Sadowski, Naoual Elftouh, Maudeline Louis, Kelley Kilpatrick, Sherilyn K. D. Houle, Jean-Philippe Lafrance

**Affiliations:** 1 Département de Pharmacologie et Physiologie, Université de Montréal, Montreal, Canada; 2 Centre de Recherche de L’Hôpital Maisonneuve-Rosemont, Montreal, Canada; 3 Faculty of Pharmacy and Pharmaceutical Sciences, University of Alberta, Edmonton, Canada; 4 Ingram School of Nursing, McGill University, Montreal, Canada; 5 School of Pharmacy, University of Waterloo, Waterloo, Canada; 6 Service de Néphrologie, CIUSSS de L’Est-de-L’Île-de-Montréal, Montreal, Canada

**Keywords:** Renin, Angiotensin aldosterone system inhibitors, Diabetic nephropathy, Diabetes mellitus, Systematic review, Meta-analysis

## Abstract

**Introduction:**

Diabetic nephropathy is the leading cause of kidney failure. Clinical practice guidelines recommend prescribing renin–angiotensin aldosterone system inhibitors (RAASi) to prevent diabetic nephropathy at any stage. We conducted this systematic review and meta-analysis to compare the effects of RAASi with placebo and other antihypertensive agents in adults with diabetes on continuous and binary kidney outcomes to provide a comprehensive review of the class effect of RAASi on several subgroups.

**Methods:**

A systematic electronic search to identify randomized clinical trials of a duration of ≥ 12 months that recruited ≥ 50 adult participants with type 1 or 2 diabetes with any stage of chronic kidney disease and proteinuria was conducted in MEDLINE, CINAHL, EMBASE, and Cochrane library with no language restriction. Studies were screened against the inclusion and exclusion criteria by two reviewers independently.

**Results:**

In this meta-analysis, evidence was drawn from 26,551 patients with diabetes from 46 studies. Our analysis shows that RAASi were better than placebo in reducing SrCr (the raw mean difference [RMD] = -13.4 μmol/L; 95%CI: -16.78; -10.01) and albuminuria levels (standardized mean difference [SMD] = -1; 95%CI: -1.57, -0.44, I^2^ = 96%). When compared to other active treatments, RAASi did not reduce SrCr (RMD = 0.03 μmol/L; 95%CI: -6.4, 6.10, I^2^ = 76%), caused a non-significant reduction of GFR levels (RMD = -1.21 mL/min; 95%CI: -4.52, 2.09, I^2^ = 86%), and resulted in modest reduction of albuminuria levels (SMD = -0.55; 95%CI: -0.95, -0.16, I^2^ = 90%). RAASi were superior to placebo in reducing the risks of kidney failure (OR = 0.74; 95%CI: 0.56, 0.97) and doubling of serum creatinine levels (SrCr; OR = 0.71; 95%CI: 0.55, 0.91), but not in promoting the regression of albuminuria (OR = 3.00; 95%CI: 0.96, 9.37). RAASi, however, were not superior to other antihypertensives in reducing the risks of these outcomes. Patients with type 2 diabetes, macroalbuminuria and longer duration of diabetes had less risk of developing kidney failure in placebo-controlled trials, while longer duration of diabetes, normal kidney function, and hypertension increased the probability of achieving regression of albuminuria in active-controlled trials.

**Conclusion:**

While our findings revealed the non-superiority of RAASi over other antihypertensives and portrayed a class effect on several subgroups of study participants, it raised a challenging question on whether RAASi deserve their place as first-line therapy in managing diabetic nephropathy.

**Supplementary Information:**

The online version contains supplementary material available at 10.1186/s12882-022-02763-1.

## Introduction

Diabetic nephropathy, a complication of diabetes, is the leading cause of kidney failure, responsible for approximately 40% of incident cases [1].

Diabetic nephropathy is characterized by hypertension, variable levels of albuminuria and a progressive loss of kidney function [2, 3]. The progression of histological and pathological changes in diabetic nephropathy are due to hyperglycemia [4]. The histological and pathological changes differ between type 1 and type 2 diabetes (T1DM, T2DM, respectively). In T1DM, hyperglycemia starts earlier hence it causes pure diabetic glomerulopathy that could be evaluated at the stage of microalbuminuria. Whereas in T2DM hyperglycemia starts later in life when kidneys were already damaged due to the long-term effects of many possible promoters of kidney injury such as aging, hypertension, and dyslipidemia. Therefore, there is a heterogenous combination of pathophysiological pathways that sustain structural changes in the kidneys of T2DM patients. Regardless of the involved mechanism, the final common pathway of diabetic nephropathy is kidney fibrosis that is caused by kidney hemodynamic and ischemic abnormalities, oxidative stress and the overactivation of the renin-angiotensin aldosterone system (RAAS) [5, 6]. Clinical practice guidelines recommend prescribing angiotensin converting enzyme (ACE) inhibitors and angiotensin receptor blockers (ARBs) which are the two major classes of renin–angiotensin aldosterone system inhibitors (RAASi) to prevent and manage diabetic nephropathy at any stage [2, 3]. The blockade of RAAS is critical to control blood volume, systemic vascular resistance and electrolyte balance [7, 8]. This results in RAASi protecting the kidneys from developing diabetic nephropathy, as well as slowing the progression of the disease [9, 10]. Therefore, these RAASi are the antihypertensive class of choice recommended for the management of patients with hypertension and DM [2, 3].

A number of meta-analyses have been published on the role of RAASi in renoprotection for patients with diabetes. The authors concluded that ACE inhibitors and ARBs are equally effective in slowing the progression of diabetic nephropathy [9–12]. However, these meta-analyses of RAASi have focused on cardiovascular and kidney outcomes, and had restricted inclusion and exclusion criteria of eligible studies and limited the included clinical trials to patient populations with one type of diabetes, a specific level of albuminuria, and/or excluding patients with advanced stages of chronic kidney disease (CKD). These findings are therefore applicable to a narrow population, and may be limited in their ability to guide clinical care and decision making for a large proportion of patients with diabetes. In addition, most of the previous meta-analyses evaluated RAASi effect on binary kidney outcomes (*e.g.* kidney failure, progression to albuminuria, mortality), and seldom provided an analysis of RAASi effect on continuous kidney outcomes (*e.g.* creatinine clearance level, albuminuria level). To address this knowledge gap we have conducted a systematic review (SR) with broader inclusion criteria to allow for conducting sub-group analysis for different variables and hence to identify their effects on kidney and other health outcomes. The objective of this systematic review and meta-analysis is to compare the effects of ACE inhibitors/ARBs with placebo and other antihypertensives in adults with diabetes on both continuous and binary kidney outcomes.

## Methods

For this systematic review, we followed the reporting guidance provided in the Preferred Reporting Items for Systematic Reviews and Meta-Analyses (PRISMA) statement (Supp 1 Table 1) [13]. The SR protocol was registered and published with PROSPERO (CRD42020149133). A brief summary of the methodology is described here, and is based on PRISMA guidelines for reporting SRs [14].

**Table 1:** Characteristics of Studies Included in the Meta-Analysis

**Study**	**Country**	**Treatment comparison**	**Treatments names**	**Setting**	**Funding**	**Albuminuria **	**Diabetes Mellitus Type**	**Stage of CKD**
Melbourne Diabetic Nephropathy Study Group 1991	Australia	RAAS inhibitors; Other Anti-HTN	Perindopril; Nifedipine	Outpatient clinic	Pharmaceutical	Microalbuminuria	Mixed (38% type 1)	Stage 1
Chan 1992	China	RAAS inhibitors; Other Anti-HTN	Enalapril; Nifedipine	Outpatient clinic	Pharmaceutical	Mixed (normoalbuminuria, microalbuminuria and macroalbuminuria)	Type2	Up to stage 4
Lacourciere 1993	Canada	RAAS inhibitors; Other Anti-HTN	Captopril; Metoprolol or HCZ	Others	Not mentioned	Mixed (normoalbuminuria and microalbuminuria)	Type2	Stage 1
Lewis 1993	USA	RAAS inhibitors; Placebo	Captopril; Placebo	Hospital	Both pharmaceutical and governmental	Not mentioned	Type1	Generally abnormal GFR
Ravid 1993 and 1995	Israel	RAAS inhibitors; Placebo	Enalapril; Placebo	Outpatient clinic	Governmental	Microalbuminuria	Type2	Stage 1
Lebovitz 1994	USA	RAAS inhibitors; Placebo	Enalapril; Placebo	Not mentioned	Both pharmaceutical and governmental	Mixed (normoalbuminuria, microalbuminuria and macroalbuminuria)	Type2	Stage 3
Viberti 1994	International	RAAS inhibitors; Placebo	Captopril; Placebo	Hospital	Pharmaceutical	Microalbuminuria	Type1	Stage 1
Agardh 1996	International	RAAS inhibitors; Other Anti-HTN	Lisinopril; Nifedipine	Not mentioned	Not mentioned	Microalbuminuria	Type2	Up to stage 3
Bakris 1996	USA	RAAS inhibitors; Other Anti-HTN	Lisinopril; Verapamil or Diltiazem; Atenolol	Outpatient clinic	Governmental	Macroalbuminuria	Type2	Generally abnormal GFR
Schnack 1996	Austria	RAAS inhibitors; Other Anti-HTN	Ramipril; Atenolol	Outpatient clinic	Not mentioned	Microalbuminuria	Type2	Stage 1
Ahmad 1997	India	RAAS inhibitors; Placebo	Enalapril; Placebo	Outpatient clinic	Governmental	Microalbuminuria	Type2	Stage 1
Chaturvedi 1997	Europe	RAAS inhibitors; Placebo	Lisinopril; Placebo	Outpatient clinic	Pharmaceutical	Mixed (normoalbuminuria, microalbuminuria and macroalbuminuria)	Type1	Not clear
Fogari 1997	Italy	RAAS inhibitors; Other Anti-HTN	Enalapril; Amlodipine	Not mentioned	Not mentioned	Microalbuminuria	Type2	Stage 1
Crepaldi 1998	Italy	RAAS inhibitors; Other Anti-HTN; Placebo	Lisinopril; Nifedipine; Placebo	Hospital and clinic	Pharmaceutical	Microalbuminuria	Type1	Stage 1
Ravid 1998	Israel	RAAS inhibitors; Placebo	Enalapril; Placebo	Outpatient clinic	Governmental	Normoalbuminuria	Type2	Stage 1
UKPDS 1998	UK	RAAS inhibitors; Other Anti-HTN	Captopril; Atenolol	Outpatient clinic	Both pharmaceutical and governmental	Mixed (normoalbuminuria, microalbuminuria and macroalbuminuria)	Type2	Stage 1
Fogari 1999	Italy	RAAS inhibitors; Other Anti-HTN	Ramipril; Nitrendipine	Not mentioned	Not mentioned	Macroalbuminuria	Type2	Generally abnormal GFR
Muirhead 1999	Canada	RAAS inhibitors; Placebo	Valsartan; Captopril; Placebo	Outpatient clinic	Pharmaceutical	Microalbuminuria	Type2	Stage 1
HOPE 2000	International	RAAS inhibitors; Placebo	Ramipril; Placebo	Hospital and clinic	Both pharmaceutical and governmental	Mixed (normoalbuminuria and microalbuminuria)	Mixed (2.3% Type 1)	Stage 1
O'Hare 2000	UK and Ireland	RAAS inhibitors; Placebo	Ramipril; Placebo	Outpatient clinic	Pharmaceutical	Microalbuminuria	Type1	Stage 1
Schrier 2000	USA	RAAS inhibitors; Other Anti-HTN	Enalapril; Nisoldipine	Not mentioned	Not mentioned	Macroalbuminuria	Type2	Up to stage 3
Tarnow 2000	Denmark	RAAS inhibitors; Other Anti-HTN	Lisinopril; Nisoldipine	Outpatient clinic	Pharmaceutical	Macroalbuminuria	Type2	Up to stage 3
Lewis 2001	International	RAAS inhibitors; Other Anti-HTN; Placebo	Irbesartan; Amlodipine; Placebo	Outpatient clinic	Pharmaceutical	Macroalbuminuria	Type2	Up to stage 5
Baines 2001	UK and Italy	RAAS inhibitors; Other Anti-HTN; Placebo	Enalapril; Nifedipine; Placebo	Hospital	Both pharmaceutical and governmental	Mixed (microalbuminuria and macroalbuminuria)	Type1	Stage 1
Bojestig 2001	Sweden	RAAS inhibitors; Placebo	Lisinopril; Placebo	Outpatient clinic	Pharmaceutical	Microalbuminuria	Type1	Not mentioned
Brenner 2001	International	RAAS inhibitors; Placebo	Losartan; Placebo	Outpatient clinic	Pharmaceutical	Macroalbuminuria	Type2	Generally abnormal GFR
Parving 2001	International	RAAS inhibitors; Placebo	Irbesartan; Placebo	Not mentioned	Pharmaceutical	Microalbuminuria	Type2	Stage 1
Kvetny 2001	Denmark	RAAS inhibitors; Placebo	Perindopril; Placebo	Not mentioned	Pharmaceutical	Normoalbuminuria	Type1	Stage 1
Baba 2001	Japan	RAAS inhibitors; Other Anti-HTN	Enalapril; Nifedipine	Outpatient clinic	Not mentioned	Mixed (normoalbuminuria and microalbuminuria)	Type2	Up to stage 3
Katayama 2002	Japan	RAAS inhibitors; Placebo	Imidapril; Captopril; Placebo	Not mentioned	Governmental	Mixed (microalbuminuria and macroalbuminuria)	Mixed (97.5% Type 1)	Stage 1
Fogari 2002	Italy	RAAS inhibitors; Other Anti-HTN	Fosinopril; Amlodipine	Not mentioned	Not mentioned	Microalbuminuria	Type2	Stage 1
Schrier 2002	USA	RAAS inhibitors; Other Anti-HTN	Enalapril; Nisoldipine	Not mentioned	Both pharmaceutical and governmental	Mixed (normoalbuminuria, microalbuminuria and macroalbuminuria)	Type2	Up to stage 3
Ahmad 2003	India	RAAS inhibitors; Placebo	Enalapril; Placebo	Outpatient clinic	Not mentioned	Microalbuminuria	Mixed (85.8% Type 1)	Stage 1
Marre 2004	International	RAAS inhibitors; Other Anti-HTN	Enalapril; Indapamide	Hospital	Pharmaceutical	Microalbuminuria	Type2	Stage 1
Jerums 2004	Australia	RAAS inhibitors; Other Anti-HTN; Placebo	Perindopril; Nifedipine; Placebo	Hospital	Both pharmaceutical and governmental	Microalbuminuria	Type2	Up to stage 2
Ruggenenti 2004	Italy	RAAS inhibitors; Other Anti-HTN; Placebo	Trandolapril; Verapamil; Placebo	Not mentioned	Both pharmaceutical and governmental	Normoalbuminuria	Type2	Stage 1
DallaVestra 2004	Italy	RAAS inhibitors; Other Anti-HTN	Ramipril; Lercanidpine	Others	Not mentioned	Microalbuminuria	Type2	Stage 1
Fogari 2005	Italy	RAAS inhibitors; Other Anti-HTN	Lisinopril; Manidipine	Outpatient clinic	Governmental	Microalbuminuria	Type2	Stage 1
Ogawa 2007	Japan	RAAS inhibitors; Other Anti-HTN	Temocapril; Candesartan; Nifedipine	Outpatient clinic	Not mentioned	Microalbuminuria	Type2	Stage 1
Makino 2008	Japan	RAAS inhibitors; Placebo	Telmisartan; Placebo	Hospital and clinic	Pharmaceutical	Microalbuminuria	Type2	Stage 1
Bilous 2009	International	RAAS inhibitors; Placebo	Candesartan; Placebo	Secondary care facility	Pharmaceutical	Normoalbuminuria	Mixed (63.6% Type 1)	Stage 1
Mauer 2009	USA and Canada	RAAS inhibitors; Placebo	Enalapril; Losartan; Placebo	Outpatient clinic	Both pharmaceutical and governmental	Normoalbuminuria	Type1	Stage 1
Haller 2011	International	RAAS inhibitors; Placebo	Olmesartan; Placebo	Secondary care facility	Pharmaceutical	Normoalbuminuria	Type2	Up to stage 3
Ruggenenti 2011	Italy and Slovenia	RAAS inhibitors; Placebo	Delapril; Placebo	Outpatient clinic	Both pharmaceutical and governmental	Mixed (normoalbuminuria and microalbuminuria)	Type2	Stage 1
Weil 2013	USA	RAAS inhibitors; Placebo	Losartan; Placebo	Not mentioned	Both pharmaceutical and governmental	Mixed (normoalbuminuria and microalbuminuria)	Type2	Stage 1
Fuchs 2016	Brazil	RAAS inhibitors; Other Anti-HTN	Losartan; Chlorthalidone/Amiloride	Secondary care facility	Governmental	Not mentioned	Type2	Not mentioned

### Research question

The clinical question of this systematic review was: In an adult who is diagnosed with T1DM or T2DM, what is the efficacy of RAASi compared with other antihypertensive medications, or with placebo on continuous kidney outcomes including eGFR, SrCr, and albuminuria levels?.

### Literature search

As there is a large number of randomized controlled trials (RCTs) and SRs on this topic, a staged approach to identify eligible RCTs was used. This approach was used successfully by other researchers [11, 15]. First, we conducted a search of relevant SRs and meta-analyses in PubMed and the Cochrane Database of Systematic Reviews. The identified SRs and meta-analyses were used to provide lists of relevant RCTs to identify studies that fit the inclusion criteria.

Next, we performed a systematic search to identify other RCTs published since the date of publication of the SRs and meta-analyses identified above. Most of the relevant meta-analyses were published around 2010, [9–12] therefore, the date limit of our systematic search was from 2010 to Jan 28, 2020. Electronic searches were conducted with the help of a medical librarian in MEDLINE, CINAHL, EMBASE, Cochrane library and the clinical trials registry at clinicaltrials.gov, with no language restriction. Search terms included generic names and Medical Subject Headings of all RAASi (including ACEIs and ARBs) combined with diabetic nephropathy and other relevant keywords as identified by the librarian (Supp 1 Table 2). Manual search of references included in relevant reviews, clinical trials and clinical practice guidelines was also conducted.

### Inclusion criteria

Studies satisfying the following criteria were included: Randomized parallel-group controlled trials of a duration of 12 months or more that recruited more than 50 adult participants (18 years or older) with T1DM or T2DM with any stage of CKD and proteinuria. The RCTs had to study the effects of RAASi on the progression of albuminuria and the progression of CKD. Progression of diabetic nephropathy was examined using the incidents of albuminuria and regression of albuminuria endpoints, and changes in urine albumin excretion levels. Progression of CKD was assessed using doubling of serum creatinine (SrCr), changes in SrCr and estimated glomerular filtration rate (eGFR).

Comparisons accepted in this SR and meta-analysis were between either ACEI or ARBs *versus* placebo or other antihypertensives including calcium channel blockers (CCBs), beta blockers (BBs), or diuretics or their combinations.

### Prespecified outcome measures

The primary outcomes of interest were continuous kidney outcomes including eGFR, SrCr levels, and albuminuria levels. Secondary outcomes were binary kidney outcomes including kidney failure, doubling of SrCr, and regression of albuminuria. Secondary outcomes also included all-cause mortality, blood pressure (BP) outcomes (diastolic and systolic BP, mean arterial BP [MAP] and the need for additional antihypertensives to control BP), and safety outcomes (the incidence of any adverse drug reactions, acute kidney injury, hyperkalemia, disruptive cough, and reasons for patients’ withdrawal from the RCTs). A list of definitions of each outcome measure is appended (Supp 1 Table 3).

### Screening and data extraction

Studies were screened against the inclusion criteria by two reviewers independently (NA and ML) using the Covidence web-based application (Veritas Health Innovation, Melbourne, Australia) [16]. Any disagreements were resolved by discussion between the two reviewers or referred to a third reviewer (JPL) when no consensus could be reached. A data extraction form was used to extract data from the eligible studies, including study and participant characteristics (sample size, age, sex, albuminuria stage, type and duration of diabetes, presence of hypertension and cardiovascular disease, smoking status, body mass index (BMI), race, and history of recent use of antihypertensives), interventions used, mean or median follow-up and outcome data. The data extraction tool was piloted on a small sample (10%) of studies by the same reviewers. One reviewer (NA) was responsible for extracting the data and the other reviewer (ML) was responsible for double-checking the entered data for accuracy. Discrepancies were resolved by consensus.

Data were extracted from studies' tables and texts reported in the main study manuscript or supplementary materials. In cases where important baseline and outcome data were not reported in tables or text, we extracted data from figures and graphs using WebPlotDigitizer which is a validated web-based application to extract numerical data from plot images [17]. Additionally, corresponding authors were contacted to seek missing or incomplete data from their studies.

Some studies provided the medians and interquartile range, and we used estimated mean of the sample equation from Luo et al. (2017), and estimated standard deviation (SD) of the sample equation from Wan et al. (2014) to calculate the means and SD, respectively using Hozo et al*.* method [18]. We calculated the effect size from the reported events numbers.

### Risk of bias assessment

Included studies' risk of bias was assessed using the Cochrane Collaboration’s risk of bias scale that addresses six domains: sequence generation, allocation concealment, blinding of participants/outcome assessors, incomplete outcome, selective outcome reporting and the source of funding [19]. Two investigators (NA and ML) were responsible for completing the assessment using the Covidence web-based application [16].

### Statistical analysis

We collectively assessed the effects of RAASi by the use of either ACEIs or ARBs on kidney outcomes by assigning trial arms of ACEIs or ARBs as the intervention group. Comparator groups were trial arms that used placebo or other antihypertensives (CCBs, BBs, diuretics or their combinations). Studies that included more than one arm including two agents of the same medication group were merged together for all outcomes.

Weighted kappa statistics were used to assess the agreement between the two reviewers for study selection. We generated descriptive statistics to provide a representation of patients included in the selected studies. We used the random effect approaches for meta-analysis of outcomes, with DerSimonian-Laird estimator for variance, to calculate the pooled effect size for each outcome because of known clinical and methodological heterogeneity of the studies. We reported the results as odds ratios (OR) using forest plots and tables.

We assessed heterogeneity between studies using I^2^ statistics with a 50% significance threshold. We used a funnel plot and Egger test to assess publication bias and Abbé plot to visually identify extreme, influential or outlier studies. We conducted different sensitivity analyses to evaluate the effect on the pooled estimate by removing the low-quality studies and removing extreme studies.

In our analysis of the continuous outcomes, we considered the difference of change from baseline between the arms of the study as the effect size for our meta-analysis except for albuminuria level, where we used standardized mean difference (SMD) as our effect size. SMD is used as an estimate of effect size when different studies measure the same outcome but in different units. Albuminuria was reported using different ways of reporting. This made SMD difficult to interpret; therefore, we used the following parameters for interpreting the size of the SMD: small, SMD = 0.2; medium, SMD = 0.5; and large, SMD = 0.8 [20]. For eGFR we considered the raw mean difference (end of the study to baseline) and the raw difference between the annual change of eGFR. All statistical assumptions used in this SR can be found in Supp 1 Table 4.

We conducted subgroup analyses to explore the effect of relevant factors for the following groups: Age groups, type and duration of diabetes, hypertension status, stage of CKD, stage of albuminuria (normoalbuminuria, microalbuminuria, macroalbuminuria), BMI category, and study duration, sample size, and year of publication on pooled effect sizes of the study, and we conducted sensitivity analysis by excluding outlier studies. We stratified the included studies based on the study duration to account for the variation of studies’ mean follow-up periods.

## Results

### Description of studies

**Fig. 1:**
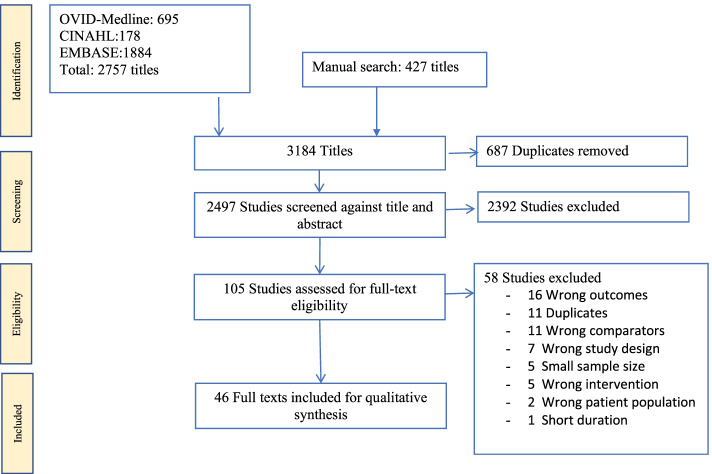
PRISMA Chart of the meta-analysis

We included 46 RCTs published between 1991 and 2016, for a total of 26,551 patients (Fig. 1). Forty-two studies had two arms, of which 22 studies conducted a comparison between RAASi and placebo. There were 38 comparisons including ACE inhibitors, mostly of enalapril (13 studies) and lisinopril (7 studies). Most ACE inhibitors were compared to active treatments (23 comparisons). All of the studies that were published in the 1990's included ACE inhibitors (18 studies). On the other hand, there were 11 studies that included ARBs, most of which were against placebo (9 comparisons) and the earliest study was published in 1999 [21] (Table 1).

The majority of trials recruited patients from outpatient clinics, and included patients with T2DM (37 studies). The trials were mostly conducted on patients with microalbuminuria at baseline (20 trials). Most of the studies (29 studies) included patients with normal kidney function (eGFR ≥ 90 mL/min), while only two studies have included patients with advanced CKD (eGFR < 30 mL/min). Four studies included patients with moderately impaired GFR (eGFR < 90 mL/min), while 3 studies did not mention if patients were excluded based on their baseline eGFR levels. The mean follow-up of the studies was 36 months (range 12–72). The mean sample size of all studies was 577 patients (range 50–5231). The average age of the patients was 51 years, while the median was 52.2 years (range 28.7–82.5). (Supp 1 Table 5).

**Fig. 2:**
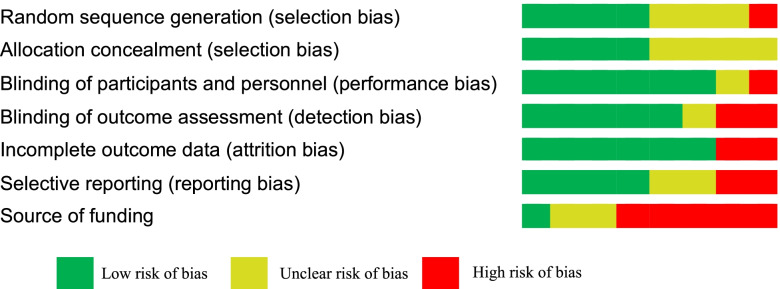
Summary of the Risk of Bias of the Included Studies

We have considered ACE inhibitors and ARBs as one class of intervention (RAASi), and therefore the comparisons carried out in our analysis included RAASi *versus* placebo, or other antihypertensives. We therefore had to exclude from our analysis comparator arms those studies that included a combination of RAASi and another antihypertensive agent in the trials with more than two intervention arms, as follows: Fogari 2002, [22] Ruggenenti 2004, [23] and Ruggenenti 2011 [24]. We also excluded studies that allowed an open-label RAASi, as in the ADVANCE trial [25]. In cases of trials that compared two different doses of an intervention, the arm with the lower and/or subtherapeutic dose was excluded from our analysis, as in the following studies: O'Hare 2000 (Ramipril 1.25 mg arm), [26] Bojestig 2001 (Lisinopril 1.25 mg), [27] Parving 2001 (Irbesartan 150 mg), [28] Makino 2008 (Telmisartan 40 mg) [29]. We combined two arms of ACE inhibitors for our analysis in one study (Katayama 2002), [30] which compared between imidapril, captopril and placebo. One study was excluded from the analysis as it was a supplemental report to a separate full-text publication [31, 32]. An overview of the meta-analysis results are available in supplementary 2 (Supp 2 Tables 1&2). The risk of bias of the included studies showed that most of the studies displayed low risk of bias in all the domains, except for the source of funding. (Fig. 2 and Supp 1 Table 8).

### Findings of the meta-analysis

#### Primary outcomes

##### Glomerular Filtration Rate – RAASi *versus* placebo

**Fig. 3:**
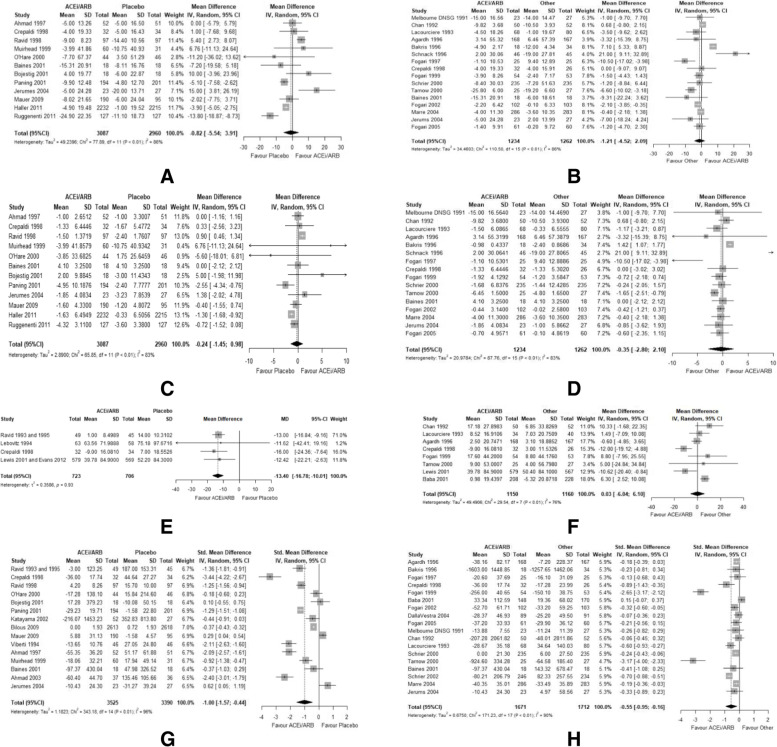
Forest plots for primary outcomes. **A** Forest plot for raw mean difference of GFR in trials comparing RAAS inhibitors versus placebo. **B** Forest plot for raw mean difference of GFR in trials comparing RAAS inhibitors *versus* other anti-hypertensives. **C** Forest plot for annual rate of change of estimated glomerular filtration rate in trials comparing RAAS inhibitors *versus* placebo. **D** Forest plot for annual rate of change of estimated glomerular filtration rate in trials comparing RAAS inhibitors *versus* other anti-hypertensives. **E** Forest plot for mean difference of serum creatinine in trials comparing RAAS inhibitors *versus* placebo. **F** Forest plot for mean difference of serum creatinine in trials comparing RAAS inhibitors *versus* other anti-hypertensives. **G** Forest plot for standardized mean difference of albuminuria levels in trials comparing RAAS inhibitors *versus* placebo. **H** Forest plot for standardized mean difference of albuminuria levels in trials comparing RAAS inhibitors *versus* other anti-hypertensives

Twelve studies [21, 24, 26–28, 33–39] (*n* = 6,047) reported the effect of RAASi compared to placebo on eGFR levels. RAASi led to a small reduction in eGFR levels (RMD = -0.82 mL/min; 95%CI: -5.54, 3.91; I^2^ = 86%; Fig. 3A), but with significant heterogeneity. The sensitivity analysis was performed by excluding one study [24] (RMD = 0.55 mL/min; 95%CI: -3.81, 4.9; I^2^ = 83%; Supp 2. Table 24C). The subgroup analysis shows that the direction of the effect size did not change among the different subgroups, except for normotensive patients, study size < 100 participants, and publication before year 2000. (Supp 2 Table 1) The effect size of RAASi on eGFR was analyzed as annual rate of change (RMD = -0.24 mL/min/year; 95%CI:-1.45, 0.98; I^2^ = 83%; Fig. 3C).

##### Glomerular Filtration Rate – RAASi *versus* other anti-hypertensives

Sixteen studies [22, 34, 35, 39–51] (n = 2,496) reported the effect of RAASi compared to active treatments on eGFR levels. RAASi led to a small reduction in eGFR levels (RMD = -1.21 mL/min; 95%CI: -4.52, 2.09; Fig. 3B). Excluding two outliers in the sensitivity analysis [40, 49] provided statistically significant results (RMD = -2.46 mL/min; 95%CI:-4.36, -0.56). All subgroups did not deviate from the pooled results except for the following subgroups: patients with T1DM, normotensive patients, mean age of patients ≥ 60 years, and mean BMI ≥ 30 kg/m^2^ (Supp 2 Table 2). RAASi did not cause a statistically significant reduction of the annual rate of change of eGFR compared to other antihypertensive agents (Annual rate of change of eGFR = -0.35 mL/min/year; 95%CI: -2.8, 2.10; Fig. 3D).

##### Serum Creatinine Levels – RAASi *versus* placebo

Four studies [31, 34, 52, 53] (*n* = 1,429) reported that RAASi resulted in a statistically significant reduction of SrCr compared to placebo (RMD = -13.4 μmol/L;95%CI: -16.78, -10.01; I^2^ = 0%; Fig. 3E) with no significant heterogeneity. These results were maintained through the subgroup analysis. (Supp 2 Table 3).

##### Serum Creatinine Levels – RAASi *versus* other anti-hypertensives

Eight studies [34, 41, 42, 47, 48, 50, 52, 54] (*n* = 2,310) reported the effect of RAASi compared to placebo on SrCr (RMD = 0.03 μmol/L; 95%CI: -6.4, 6.10; I^2^ = 76%; Fig. 3F). Subgroup analysis showed higher mean difference levels of SrCr in favor of the active treatments in studies that lasted ≤ 2 years, while the opposite was observed in longer studies of more than 2 years duration (4.38; 95%CI: -0.66, 9.42 *versus* -6.36; 95%CI: -14.46, 1.75), respectively. (Supp 2 Table 4).

##### Albuminuria Levels – RAASi *versus* placebo

Fifteen studies [21, 26–28, 30, 31, 34–39, 55–57] (*n* = 6,915) reported the effect of RAASi compared to placebo on albuminuria levels. The meta-analysis showed a large difference in the effect of RAASi in reducing albuminuria levels (SMD = -1; 95%CI: -1.57, -0.44; Fig. 3G). The sensitivity analysis was performed by excluding seven outlier studies[34–37, 55–57] (SMD = -0.75; 95%CI: -1.14, -0.37; I^2^ = 85%), indicating a medium effect size. These results were maintained through the subgroup analysis. (Supp 2 Table 5).

##### Albuminuria Levels – RAASi *versus* other anti-hypertensives

Eighteen studies [22, 34, 35, 39–48, 50, 51, 54, 58, 59] (*n* = 3,383) reported the effect of RAASi compared to active treatments on albuminuria levels. (Fig. 3H) We found a moderate difference in the effect of RAASi in reducing albuminuria levels (SMD = -0.55; 95%CI: -0.95, -0.16). The sensitivity analysis was performed by excluding three outlier studies [41, 50, 54] (SMD = -0.31; 95%CI: -0.44, -0.18). Subgroups of T1DM and macroalbuminuria had even lower SMD in the same direction of the pooled SMD. On the other hand, subgroups of microalbuminuria, no CKD, mean age ≥ 60 years, and sample size ≥ 100 participants, had lower SMDs compared to the other subgroups (Supp 2 Table 6).

#### Secondary outcomes

RAASi reduced the risk of kidney failure and doubling of SrCr when compared to placebo (OR = 0.74; 95%CI: 0.56, 0.97 & OR = 0.71; 95%CI: 0.55, 0.91; respectively). The subgroup analysis presents a homogenous effect of different subgroups, all in favor of RAASi. Additionally, RAASi increased the probability of achieving regression of albuminuria compared to placebo (OR = 3.00; 95%CI: 0.96, 9.37). All subgroups agreed on the favorable effect of RAASi in inducing the regression of albuminuria, and certain subgroups presented statistically significant outcomes, including the subgroups of patients with hypertension, no CKD and BMI < 30 kg/m^2^. Further details on the secondary kidney outcomes, all-cause mortality, blood pressure, and adverse effects are provided in supplementary 2.

## Discussion

In this meta-analysis, evidence was drawn from 26,551 patients with diabetes from 46 studies on the effect of RAASi on continuous and binary kidney outcomes. This meta-analysis provided interesting findings on the effects of RAASi on some important continuous outcomes in comparison with placebo or other antihypertensives. RAASi were better than placebo in reducing SrCr and albuminuria. Estimated GFR was slightly increased by RAASi compared to placebo after performing a sensitivity analysis. When compared to active treatments, RAASi resulted in a modest increase of SrCr, led to eGFR decline, and resulted in modest reduction of albuminuria levels. Our analysis shows that the RAASi class was superior to placebo in reducing the risks of kidney failure and doubling of SrCr levels, but not in promoting the regression of albuminuria. RAASi, however, were not superior to other antihypertensive agents in reducing the risks of these kidney outcomes or all-cause mortality. Despite some key differences in the selection criteria (Supp 1 Table 6), the findings of our meta-analysis are consistent with previously published meta-analyses.

What distinguishes our meta-analyses from earlier reviews is the inclusion criteria of clinical trials, as well as our analysis of continuous kidney outcomes.(Supp 1 Table 6) We excluded RCTs with sample size of less than 50 participants to exclude small-size effects on the analysis. We performed subgroup analysis based on sample size to further isolate small-size effect of the studies of less than 100 participants. A duration of 12 months or more was a key inclusion criterion to help study the long-term effects of RAASi. Unlike some earlier meta-analyses, we did not limit our analysis to one type of diabetes or to a specific degree of kidney function or albuminuria, which allowed us to perform a variety of subgroup analyses.

This meta-analysis provided noteworthy findings about the effect of RAASi in each subgroup of patients. We have studied RAASi effects on each kidney outcome in different subgroups of study participants. The analysis showed that specific subgroups of patients had better outcomes with RAASi. Patients with T2DM, macroalbuminuria and an average duration of diabetes more than or equal 10 years had less risk to develop kidney failure in placebo-controlled trials, while longer duration of diabetes, normal kidney function, and hypertension increased the probability to achieve regression of albuminuria in active-controlled trials. Type 1 diabetes and hypertensive patients had higher rates of regression of albuminuria in placebo-controlled trials. As these findings suggest, the type and the duration of diabetes as well as other characteristics can influence the response to interventions on some kidney outcomes, which highlights the importance to consider each patient’s medical history when deciding on starting a treatment for them. These findings point to the need to direct more research initiatives on exploring patients’ characteristics that can predict who would benefit most from each intervention, including the broadening of inclusion criteria in studies, and conducting studies powered to look at divergent subgroups. The interpretation of subgroup analyses should be performed with caution, due to some inherited limitations, majorly because they are observational in nature although being derived from randomized trials [60].

The latest version of the Kidney Disease Improving Global Outcomes (KDIGO) clinical practice guidelines for diabetes management in CKD patients [61] were published in late 2020, and it recommends using RAASi as first-line antihypertensives for patients with diabetes, hypertension and albuminuria. These recommendations were based on four placebo-controlled landmark trials of ARBs on patients with different levels of albuminuria [28, 29, 52, 62]. This recommendation is concordant with those of other guidelines, [28, 29, 62] yet it raises some doubts about the robustness of the evidence behind it. The KDIGO guideline supports its recommendation with evidence from trials of ARBs only. Nevertheless, our analysis on continuous outcomes provides moderate evidence on RAASi’s ability to reduce albuminuria levels more than active-treatments. Cativo et al*. *[63] reached a similar conclusion, and highlighted that although the effect is statistically significant, the clinical effect is small. In summary, the evidence behind promoting RAASi as the leading class in protecting the diabetic kidneys may not be as robust as commonly believed. The findings of this study suggest that the most important factor for preventing and managing diabetic nephropathy is lowering BP levels, which could be of higher significance than the class of the antihypertensive used to lower BP.

Protecting the diabetic kidneys is not exclusive to antihypertensives, as some novel classes claimed their positions in the competition towards protecting diabetic patients from kidney disease using different mechanisms. For example, the mineralocorticoid receptor antagonist (finerenone) is being evaluated in a large RCT (FIDELIO-DKD), with some preliminary promising results [64]. The new antihyperglycemic agents from the sodium‐glucose co‐transporter‐2 (SGLT2) inhibitors class have also shown protective effects against progression of CKD, with reductions of mortality rates when used in combination with RAASi [65]. Sacubitril/valsartan have shown preservation effects of kidney function in older patients with heart failure, and its role in the management of diabetic nephropathy is to be evaluated [66]. Taking the collective adverse events of these agents into consideration, the prescriber today has more options to consider to reduce the progression of diabetic kidney disease. A prescription that combines these agents with a proper antihypertensive could be viewed as the recipe of kidney protection in patients with diabetes. Nevertheless, more research studies need to be carried out to prove the safety and efficacy of such combinations.

This meta-analysis sheds light on the full spectrum of RAASi effects on kidney outcomes in patients with diabetes, by studying its efficacy on both continuous and binary outcomes. It provides a comprehensive review of the class effect on several subgroups of study participants, which was facilitated by the broad inclusion criteria. While we were attempting to answer a research question on RAASi comparative efficacy, our study raised a challenging question on the role of RAASi in preventing and managing diabetic nephropathy and whether it deserves its place as a first-line therapy in the clinical practice guidelines. The analysis' protocol was initially designed to include studies that reported other relevant kidney outcomes including urinary albumin concentration, albumin creatinine ratio, fractional albumin excretion, and kidney deaths. Therefore, the initial number of included studies were 53 trials. However, there was a very limited number of studies that collectively reported these outcomes, which lead to insufficient data to perform meaningful analysis of these outcomes. Therefore, the final number of included studied was 46.

A few limitations of this meta-analysis should be considered when interpreting and applying its findings. The analysis of the continuous outcomes was performed using a number of statistical assumptions.(Supp 1 Table 4) Another limitation is the degree of heterogeneity between the RCTs that were used to determine the change in the continuous outcomes. These RCTs were published across more than 20 years with variable methodological approaches and reporting qualities that resulted in methodological heterogeneity. The included studies shared a wide range of participant characteristics due to our broad inclusion criteria which resulted in population heterogeneity. The performance of subgroup analysis and sensitivity analysis helped mitigate the effect of this type of heterogeneity. The mean follow-up of included studies ranged between 12 and 72 months. Therefore, we conducted subgroup analyses for each outcome to account the difference in the duration of follow-up between studies. (Supp 2, tables 3–18).

It is noteworthy to mention that most of the RCTs were not powered to detect the changes in the continuous kidney outcomes because these were not primary outcomes. We have calculated the effect size from the reported events numbers because of the heterogeneity in reporting effect sizes between studies. We did not analyze data presented in composite outcomes, because of the inconsistency of the trials in reporting these outcomes as the same composite. However, we analyzed data for each single outcome separately.

## Conclusion

This systematic review and meta-analysis identified 46 studies, andshowed that RAASi class was better than placebo in reducing SrCr and albuminuria levels. When compared to other active treatments, RAASi did not reduce SrCr levels, caused a non-significant reduction of eGFR, and resulted in modest reduction of albuminuria levels. These results were reported with considerable statistical heterogeneity. As for binary outcomes, RAASi were superior to placebo but not the other antihypertensive agents in reducing the risks of kidney failure and doubling of SrCr..While our findings revealed the non-superiority of RAASi over other antihypertensives it raised some doubts about the robustness of evidence behind placing RAASi as first-line therapy in managing diabetic nephropathy.

## Supplementary Information

**Additional file 1:** **Supplementary 1.**

**Additional file 2:** **Supplementary 2.**

**Additional file 3:** **Supplementary 3**.

## Data Availability

All data generated or analyzed during this study are included in this article and its additional files.

## Abbreviations

RAASi: Renin–angiotensin aldosterone system inhibitors
DM: Diabetes mellitus
SrCr: Serum creatinine
OR: Odd ratio
95%CI: 95% Confidence interval
RMD: Raw mean difference
SMD: Standardized mean difference
ACE: Angiotensin converting enzyme
ARBs: Angiotensin receptor blockers
CKD: Chronic kidney disease
SR: Systematic review
T1DM: Type 1 diabetes mellitus
T2DM: Type 2 diabetes mellitus
RCTs: Randomized controlled trials
eGFR: Estimated glomerular filtration rate
CCBs: Calcium channel blockers
BB: Beta blockers
BP: Blood pressure
MAP: Mean arterial BP
BMI: Body mass index
SD: Standard deviation
