# Supplementary material for: The effect of renin–angiotensin–aldosterone system inhibitors on continuous and binary kidney outcomes in subgroups of patients with diabetes: a meta-analysis of randomized clinical trials
Source: BMC Nephrol. 2022 Apr 28;23:161. doi: 10.1186/s12882-022-02763-1 (PMC9052620; doi:10.1186/s12882-022-02763-1)
Supplement: Supplementary file 1 — Additional file 1: Supplementary 1. [file 12882_2022_2763_MOESM1_ESM.pdf]

The Effect of Renin-Angiotensin-Aldosterone System Inhibitors on Binary and Continuous Renal Outcomes in Subgroups of Patients with Diabetes: An Extensive Meta-Analysis

Supplement 1

Table of Contents

SUPP 1. TABLE 1: PRISMA CHECKLIST 2

SUPP 1. TABLE 2: SEARCH STRATEGY IN MEDLINE 4

SUPP 1. TABLE 3: DEFINITIONS OF BINARY OUTCOMES: 8

SUPP 1. TABLE 4: THE ANALYSIS OF THE CONTINUOUS OUTCOMES USING A NUMBER OF STATISTICAL ASSUMPTIONS 9

SUPP 1. TABLE 5: CHARACTERISTICS OF STUDY POPULATIONS IN INCLUDED RANDOMIZED CONTROLLED TRIALS 11

SUPP 1. TABLE 6: COMPARISON BETWEEN OUR AND PREVIOUS META-ANALYSES 13

SUPP 1. TABLE 7: INTER-RATER RELIABILITY TO ASSESS FOR THE AGREEMENT BETWEEN THE TWO REVIEWERS FOR STUDY SELECTION USING WEIGHTED KAPPA STATISTICS 15

SUPP 1. TABLE 8: STUDIES' RISK OF BIAS USING THE COCHRANE COLLABORATION'S RISK OF BIAS SCALE 16

SUPP 1. TABLE 9: REPORTED BINARY RENAL OUTCOMES IN THE INCLUDED TRIALS (33 TRIALS) 18

SUPP 1. TABLE 10: REPORTED CONTINUOUS RENAL OUTCOMES IN THE INCLUDED TRIALS (39 TRIALS) 20

SUPP 1. LIST 1: A LIST OF THE CITATIONS OF THE INCLUDED CLINICAL TRIALS: 46 INCLUDED RCT PRESENTED IN 50 PUBLISHED ARTICLES 22

Supp 1. Table 1: PRISMA Checklist

Section/topic	#	Checklist item	Reported on page #
**TITLE**			
Title	1	Identify the report as a systematic review, meta-analysis, or both.	1
**ABSTRACT**			
Structured summary	2	Provide a structured summary including, as applicable: background; objectives; data sources; study eligibility criteria, participants, and interventions; study appraisal and synthesis methods; results; limitations; conclusions and implications of key findings; systematic review registration number.	2
**INTRODUCTION**			
Rationale	3	Describe the rationale for the review in the context of what is already known.	3
Objectives	4	Provide an explicit statement of questions being addressed with reference to participants, interventions, comparisons, outcomes, and study design (PICOS).	3
**METHODS**			
Protocol and registration	5	Indicate if a review protocol exists, if and where it can be accessed (e.g., Web address), and, if available, provide registration information including registration number.	3
Eligibility criteria	6	Specify study characteristics (e.g., PICOS, length of follow-up) and report characteristics (e.g., years considered, language, publication status) used as criteria for eligibility, giving rationale.	4
Information sources	7	Describe all information sources (e.g., databases with dates of coverage, contact with study authors to identify additional studies) in the search and date last searched.	4
Search	8	Present full electronic search strategy for at least one database, including any limits used, such that it could be repeated.	4
Study selection	9	State the process for selecting studies (i.e., screening, eligibility, included in systematic review, and, if applicable, included in the meta-analysis).	5

Data collection process	10	Describe method of data extraction from reports (e.g., piloted forms, independently, in duplicate) and any processes for obtaining and confirming data from investigators.	5
Data items	11	List and define all variables for which data were sought (e.g., PICOS, funding sources) and any assumptions and simplifications made.	5
Risk of bias in individual studies	12	Describe methods used for assessing risk of bias of individual studies (including specification of whether this was done at the study or outcome level), and how this information is to be used in any data synthesis.	6
Summary measures	13	State the principal summary measures (e.g., risk ratio, difference in means).	6
Synthesis of results	14	Describe the methods of handling data and combining results of studies, if done, including measures of consistency (e.g., I^2) for each meta-analysis.	6

Section/topic	#	Checklist item	Reported on page #
Risk of bias across studies	15	Specify any assessment of risk of bias that may affect the cumulative evidence (e.g., publication bias, selective reporting within studies).	6
Additional analyses	16	Describe methods of additional analyses (e.g., sensitivity or subgroup analyses, meta-regression), if done, indicating which were pre-specified.	6
**RESULTS**			
Study selection	17	Give numbers of studies screened, assessed for eligibility, and included in the review, with reasons for exclusions at each stage, ideally with a flow diagram.	7
Study characteristics	18	For each study, present characteristics for which data were extracted (e.g., study size, PICOS, follow-up period) and provide the citations.	7
Risk of bias within studies	19	Present data on risk of bias of each study and, if available, any outcome level assessment (see item 12).	7
Results of individual studies	20	For all outcomes considered (benefits or harms), present, for each study: (a) simple summary data for each intervention group (b) effect estimates and confidence intervals, ideally with a forest plot.	7-11
Synthesis of results	21	Present results of each meta-analysis done, including confidence intervals and measures of consistency.	7-11
Risk of bias across studies	22	Present results of any assessment of risk of bias across studies (see Item 15).	7-11
Additional analysis	23	Give results of additional analyses, if done (e.g., sensitivity or subgroup analyses, meta-regression [see Item 16]).	7-11
**DISCUSSION**			

Summary of evidence	24	Summarize the main findings including the strength of evidence for each main outcome; consider their relevance to key groups (e.g., healthcare providers, users, and policy makers).	11
Limitations	25	Discuss limitations at study and outcome level (e.g., risk of bias), and at review-level (e.g., incomplete retrieval of identified research, reporting bias).	13
Conclusions	26	Provide a general interpretation of the results in the context of other evidence, and implications for future research.	14
**FUNDING**			
Funding	27	Describe sources of funding for the systematic review and other support (e.g., supply of data); role of funders for the systematic review.	1

From: Moher D, Liberati A, Tetzlaff J, Altman DG, The PRISMA Group (2009). Preferred Reporting Items for Systematic Reviews and Meta-Analyses: The PRISMA Statement. PLoS Med 6(7): e1000097. doi:10.1371/journal.pmed1000097

For more information, visit: **www.prisma-statement.org**.

Supp 1. Table 2: Search Strategy in Medline

Concepts	#	Searches	Results
	Database(s): **All Ovid MEDLINE(R)** 1946 to Present, searched on Jan 28 2020		
	**1**	**exp Angiotensin-Converting Enzyme Inhibitors/**	**43109**
	2	(((Angiotensin adj1 Converting Enzyme) or ACE) adj (Inhibit* or Antagonist?)).tw,kw.	34846

	3	(alacepril or benazepril or benazeprilat or Captopril or ceronapril or Cilazapril or cilazaprilat or delapril or Enalapril or Enalaprilat or Fosinopril or fosinoprilat or gemopatrilat or imidapril or imidaprilat or libenzapril or Lisinopril or moexipril or omapatrilat or Perindopril or perindoprilat or Quinapril or quinaprilat or Ramipril or ramiprilat or rentiapril or sampatrilat or spirapril or temocapril hydrochloride or Teprotide or trandolapril or zofenopril or capoten or lopirin or renitec or renitek or vasotec or fosinorm or monopril or prinivil or Zestril or moexipril or Accupril or triatec or tritace or inhibace or trandolapril).tw,kw.	26345
	4	exp Angiotensin Receptor Antagonists/	23177
	5	(Angiotensin adj2 Receptor adj1 (Antagonist? or inhibit* or blocker?)).tw,kw.	12205
	6	(ARB? or sartan? or azilsartan or candesartan or candesartan or enoltasosartan or eprosartan or Irbesartan or Losartan or olmesartan or saprisartan or Telmisartan or Valsartan or Fimasartan or Forasartan cozaar or aprovel or avapro or benicar or olmetec or micardis or pritor or diovan or Atacand or Edarbi or Teveten).tw,kw.	22789
A	7	1 or 2 or 3 or 4 or 5 or 6	80975
B1	8	DIABETIC NEPHROPATHIES/pc or Proteinuria/pc or Albuminuria/pc	3914
	9	DIABETIC NEPHROPATHIES/ or ((Proteinuria/ or Albuminuria/) and (exp diabetes mellitus/ or diabet*.tw,kw.))	30988
	10	((diabet* or nodular or Intracapillary or Intra-capillary) adj glomerulosclerosis).tw,kw.	733
	11	diabet* glomerulopath*.tw,kw.	333
	12	(diabetic adj (nephropath* or nephrosclerosis or kidney disease?)).tw,kw.	19316
	13	((Proteinuria? or Albuminuria? or microalbuminuria? or macroalbuminuria? or NephroprotectI* or Nephro-protectI* or renal loss* or kidney loss*) and diabet*).tw,kw.	16830

	14	(((micro or macro) adj albuminaria?) and diabet*).tw,kw.	0
	15	(diabetic adj (nephropath* or nephrosclerosis or kidney disease?)).tw,kw.	19316
B2	16	9 or 10 or 11 or 12 or 13 or 14 or 15	41965
	17	(KIDNEY FAILURE, CHRONIC/ or RENAL INSUFFICIENCY, CHRONIC/ or GLOMERULAR FILTRATION RATE/) and (Diabetes Mellitus, Type 1/ or Diabetes Mellitus, Type 2/ or diabet*.tw,kw.)	19383
	18	((((chronic or end) adj1 stage adj1 (renal or kidney) adj1 (disease? or insufficienc* or failure? or impairment? or dysfunction?)) or (ren* adj1 (outcome or endpoint or protec*)) or renoprotec* or nephropath* or CKD or ESRD) and (Diabet* or hyperglycemi* or glycemi*)).tw,kw.	36217
	19	((endstage adj1 (renal or kidney) adj1 (disease? or insufficienc* or failure? or impairment? or dysfunction?)) and (Diabet* or hyperglycemi* or glycemi*)).tw,kw.	112
B3	20	17 or 18 or 19	44447
	21	exp RANDOMIZED CONTROLLED TRIAL/	500128
	22	(Randomized Controlled Trial or Controlled Clinical Trial or Pragmatic Clinical Trial or Equivalence Trial or Clinical Trial, Phase III).pt.	591222
	23	Randomized Controlled Trial/ or exp Randomized Controlled Trials as Topic/ or "Randomized Controlled Trial (topic)"/ or Controlled Clinical Trial/ or exp Controlled Clinical Trials as Topic/ or "Controlled Clinical Trial (topic)"/ or Randomization/ or Random Allocation/ or Double-Blind Method/ or Double Blind Procedure/ or Double-Blind Studies/ or Single-Blind Method/ or Single Blind Procedure/ or Single-Blind Studies/ or Placebos/ or Placebo/ or Control Groups/ or Control Group/	827481
	24	(random* or sham or placebo*).ti,ab,hw,kf,kw.	1433944
	25	((singl* or doubl*) adj (blind* or dumm* or mask*)).ti,ab,hw,kf,kw.	230001
	26	((tripl* or trebl*) adj (blind* or dumm* or mask*)).ti,ab,hw,kf,kw.	912

	27	(control* adj3 (study or studies or trial* or group*)).ti,ab,kf,kw.	934605
	28	(Nonrandom* or non random* or non-random* or quasi-random* or quasirandom*).ti,ab,hw,kf,kw.	41825
	29	allocated.ti,ab,hw.	62215
	30	((open label or open-label) adj5 (study or studies or trial*)).ti,ab,hw,kf,kw.	32660
	31	((equivalence or superiority or non-inferiority or noninferiority) adj3 (study or studies or trial*)).ti,ab,hw,kf,kw.	7559
	32	(pragmatic study or pragmatic studies).ti,ab,hw,kf,kw.	362
	33	((pragmatic or practical) adj3 trial*).ti,ab,hw,kf,kw.	4606
	34	((quasiexperimental or quasi-experimental) adj3 (stuzy or studies or trial*)).ti,ab,hw,kf,kw.	1510
	35	(phase adj3 (III or "3") adj3 (study or studies or trial*)).ti,hw,kf,kw.	26990
C	36	21 or 22 or 23 or 24 or 25 or 26 or 27 or 28 or 29 or 30 or 31 or 32 or 33 or 34 or 35	2061009
A AND B1 AND C,	37	7 and 8 and 36	461
(A AND (B2 OR B3) AND C) Not 1st set	38	(7 and (16 or 20) and 36) not 37	1638
1st set in last 10 yrs	39	limit 37 to yr="2010 -Current"	127
2nd set in last 10 yrs	40	limit 38 to yr="2010 -Current"	568

Supp 1. Table 3: Definitions of primary and secondary outcomes:

Outcome	Definition
**Primary Outcomes**	
Kidney failure	The chronic need to renal replacement therapy/dialysis or the need for kidney transplant
Doubling of serum creatinine	The doubling of serum creatinine value from its baseline value that was measured at the start of the trial
Regression of albuminuria	The improvement and change of albuminuria from a higher category of albuminuria at baseline to a lower category
Glomerular filtration rate	The measure of the flow rate of filtered fluid through the kidney glomerular capillaries per unit time to estimate the kidney function and determines the stage of kidney disease. It is calculated using Cockcroft-Gault formula, The Modification of Diet in Renal Disease (MDRD) Study equation and the Chronic Kidney Disease Epidemiology Collaboration (CKD-EPI) equation. Glomerular filtration rate depends on blood creatinine level, age, body weight and gender.
Serum creatinine levels	A measure of creatinine levels in the blood, that helps in determining kidney function and the stage of kidney disease.
Albuminuria levels	It is the measurement of albumin in urine that helps determine the level of diabetic nephropathy, as follows: A1 (normoalbuminuria) <30mg/g; A2 (microalbuminuria) 30-300 mg/g; A3 (macroalbuminuria) >300 mg/g.
**Secondary Outcomes**	

All-cause mortality	The total number of deaths from all causes for the study population during the study time period.
Need for additional antihypertensives	The number of patients that failed to achieve the target blood pressure within the specified time period according to the clinical trial protocol, and therefore they require to receive an additional antihypertensive medication other than the trial's randomized intervention(s) to achieve the target blood pressure.
Cases with disruptive cough	The number of patients who developed a disruptive cough that had started after the commence of the trial, and which could not be associated with a specific diagnosis.

Supp 1. Table 4: The analysis of the continuous outcomes using a number of statistical assumptions

• **Description of statistical analysis for continuous outcomes**
• We considered the difference of change from baseline between the arms of the study as the effect size for our meta analysis except for Alb24h/AER : the mean difference between the change from baseline in the intervention group and the change from baseline in the control group (placebo or other anti-HTN drugs).<br>• For GFR, in addition to the mean difference of the change from baseline, we considered the difference in the annual rate of decline between the arms of the study.<br>• For Alb24h/AER outcome, we used the standardized mean difference as our effect size.

- If the study includes two arms in the same category, we combined the data for the two arms (ex: two arms ACEi/ARB or two arms Other Anti-HTN);
    - Mean =(n1*mean1+n2*mean2)/n1+n2
    - SD=√ [(n1-1)var1+(n2-1)*var2/(n1+n2-2)]
- If the study reported data by subgroup, we combined the results of subgroups (ex: data presented by hypertension status);
- If the study reported the change from baseline for each arm and its SD, we considered these data directly into the calculation of the effect size
- If the study reported data at baseline and at the end of the study, we calculated the change from baseline and its SD;
    - Mean difference = Mean post − Mean pre; for each arm in the study
    - SD = √[(SDpre)2 + (SDpost)2 − 2 × corr(pre, post) × SDpre × SDpost]
- We have considered a conservative estimate for the correlation coefficient: corr(pre, post) =0.4 (Cochrane and Shuyan 2015).
- If the mean at baseline or at the end of the study is missing and the change from baseline is not reported and could not be inferred from the study results, we excluded the study from the comparison (eg. Lewis 2001 for AER).
- We used random-effects meta-analysis for all outcomes and subgroup analysis.
- Publication bias was assessed with visual inspection of funnel plots.
- We tested Heterogeneity using chi-square and I^2 tests with a significance level of 0.05 for the chi-square test.
- We have done a sensitivity analysis by excluding the outlier studies from the meta analysis.
- We conducted different subgroup analysis (eg. type of diabetes, year of publication…).

- **Estimation of missing SDs**

    - If we have SD missing and confidence intervals we estimate SD using the formula SD=(UCL- LCL)/3.92.
    - If we have SD missing and confidence interval using SEM, we use the following formula racine(n)*(UCL- LCL)/3.92;
    - If we have the median and IQR we use the formula IQR/1.35.
    - If we have the median and the range, we calculated the SD by dividing the range by 4 if n <70 and by 6 if not.
    - If we have SD at the end of the study missing, then we consider it equal to SD baseline.

- **Converting the geometric mean to the arithmetic mean**
- This is the case for some studies reporting GFR, ALB24h and AER as geometric mean.
- We transformed the geometric mean and its antilog or confidence intervals to log transformed data.
- We used Higgings formulas (Higgings 2008, method 1) to calculate the arithmetic means and their SDs.

- **Calculation of the annual rate of decline**
- If the study reported the annual rate of decline, we used it directly.
- If the study reported a monthly decline, we calculate the annual decline by multiplying it by 12.

- If the study reported the baseline and the end of study means for each arm, we calculate the annual decline by dividing the difference from baseline by the study duration.
- If the study reported different monthly declines during the study, we used this information to calculate the total decline from the start of the study and divided it by the study duration.

Supp 1. Table 5: Characteristics of Study Populations in included Randomized controlled Trials

								Albuminuria (n)			Race (n)					Smoking status		
**Study, Year**	**Total sample size**	**Male (%)**	**Mean age (years)**	**Type1 Diabetes (n)**	**Type2 Diabetes (n)**	**Hypertension (%)**	**CVD (%)**	**Normo**	**Micro**	**Macro**	**White**	**Black**	**Hispanic**	**Asian**	**O ther**	**Current**	**Former**	**Never**
Melbourne Diabetic Nephropathy Study Group 1991	50	70%	54.1	19	31			0	50	0								
Chan 1992	102	40%	43.1	0	102	100%	0%	44	36	22	0	0	0	102				
Lacourciere 1993	74	57%	57.5	0	74	100%	0%	53	21	0	74	0	0	0				
Lewis 1993	409	53%	41.1	409	0	76%					364	30	0	0				
Ravid 1993 and 1995	94	45%	52.7	0	94	0%		0	94	0								
Lebovitz 1994	121		30	0	121	100%		37	38	46								
Viberti 1994	92	55%	46.5	92	0	0%	0%	0	92	0						49		
Agardh 1996	335	71%	46.5	0	335	100%		2	333	0								
Bakris 1996	52	38%	53.3	0	52	100%	0%	0	0	52	24	28	0	0				
Schnack 1996	91	30%	63.5	0	91	100%		0	91	0								
Ahmad 1997	103	58%	42.6	0	103	0%	0%	0	103	0	0	0	0	103				
Chaturvedi 1997	530	61%	36.8	530	0	0%	0%	440	79	6								
Fogari 1997	50	100%	49.2	0	50	100%		0	50	0								
Crepaldi 1998	92	68%	37.8	92	0	0%	12%	0	92	0						56		
Ravid 1998	194	39%	45.9	0	156	0%	0%	156	0	0						29		
UKPDS 1998	758	54%	59.1	0	758	100%	0%	527	96	18	651	62		39		171	294	281
Fogari 1999	52	98%	48.9	0	51	98%	0%	0	0	51								

								Albuminuria (n)			Race (n)					Smoking status		
**Study, Year**	**Total sample size**	**Male (%)**	**Mean age (years)**	**Type1 Diabetes (n)**	**Type2 Diabetes (n)**	**Hypertension (%)**	**CVD (%)**	**Normo**	**Micro**	**Macro**	**White**	**Black**	**Hispanic**	**Asian**	**O ther**	**Current**	**Former**	**Never**
Muirhead 1999	91	74%	57.1	0	91	41%		0	91	0	83	1	0	3				
HOPE 2000	3577	63%	60.5	81	3496	56%	69%	2437	1140	0						544		
O'Hare 2000	134	48%	46.3	134	0	0%	0%	0	134	0								
Schrier 2000	470	67%	59.8	0	470	100%	51%			82								
Tarnow 2000	48	67%	53	0	50	100%		0	0	50	50	0	0	0		27		
Lewis 2001	1715	66%	49.4	0	1715	100%	29%	0	0	1715	1242	228	83	85				
Baines 2001	54	63%	31.1	54	0	0%	0%	0								22		
Bojestig 2001	55	75%	38	55	0	0%	0%	0	55	0								
Brenner 2001	1513	63%	49.1	0	1513	96%	21%	0	0	1513	736	230	276	252		277		
Parving 2001	590	68%	57.2	0	590	100%	27%	0	590	0	574					110	223	257
Kvetny 2001	89	30%	51	89	0	0%	0%	89	0	0								
Baba 2001	436	50%	62.6	0	436	100%		159	159	0	0	0	0	436		72		
Katayama 2002	81	35%	31.8	79	3	17%	0%				0	0	0	79				
Fogari 2002	309	57%	55.6	0	309	100%	0%	0	309	0								
Schrier 2002	480	55%	60	0	480	0%	24%	317	111	52	350	34	82			62	220	198
Ahmad 2003	85	45%	28.7	73	12	0%	0%	0	73	0				72				
Marre 2004	570	23%	57.6	0	569	100%		0	569	0						28	46	126
Jerums 2004	77	64%	54.7	0	77	0%	0%	0	77	0						24		
Ruggenenti 2004	904	70%	82.5	0	1204	133%		1204	0	0						144	362	698
DallaVestra 2004	180	73%	62	0	180	100%	4%	0	180	0								
Fogari 2005	121	49%	51.1	0	121	100%		0	121	0						0		
Ogawa 2007	92	47%	61.5	0	92	100%		0	92	0	0	0	0	92				
Makino 2007	514	73%	60	0	514	68%	0%	0	514	0	0	0	0	514	0	0	0	0
Bilous 2009	5231	54%	36.3	3326	1905	23%		5231	0	0	5076					1145	492	3594
Mauer 2009	285	15%	38.8	285	0	0%		285	0	0	279	0	0	0				

								Albuminuria (n)			Race (n)					Smoking status		
**Study, Year**	**Total sample size**	**Male (%)**	**Mean age (years)**	**Type1 Diabetes (n)**	**Type2 Diabetes (n)**	**Hypertension (%)**	**CVD (%)**	**Normo**	**Micro**	**Macro**	**White**	**Black**	**Hispanic**	**Asian**	**O ther**	**Current**	**Former**	**Never**
Haller 2011	4447	46%	53.9	0	4447		33%	4447	0	0	4447	0	0	0		832	905	2708
Ruggenenti 2011	380	65%	60.2	0	380	100%		328	52	0						49	150	181
Weil 2013	169	29%	52.3	0	169	12%	0%	91	78	0	0	0	0	0	0	0	0	0
Fuchs 2016	655	51%	52	0	655	100%					403	252	0	0		48	310	208

Supp 1. Table 6: Comparison between Our and Previous Meta-analyses

	Our MA	Lopez 2016	Wu 2013	Vejakama 2012	Casas 2005	Palmer 2015	Coleman 2019
Inclusion criteria	MA of 46 RCTs that recruited more than 50 adults participants comparing the renal effects of RAASi vs placebo or other classes of active Treatment in patients with diabetes, with a follow-up >= 12 months	Network MA of 71 RCT comparing the renal and CVD effects single or combination of RAASI vs placebo or other classes of active Treatment in patients with diabetes, better than=18 years, with a follow-up >= 12 months.	Network MA of 63 RCTs comparing the effects of any single or combination of antihypertensive drugs with placebo or other classes of active treatments in patients with diabetes >=18 years, with a follow-up >= 12 months.	Network MA of 28 RCT comparing the effects of RAASI vs placebo or other classes of active treatment in patients with T2DM, >=18 years. of any study duration .	MA of 127 RCTs comparing the effects of any single or combination of antihypertensive drugs with placebo or vs active treatment in patients with diabetes or no diabetes with a follow-up >= 12 months.	Network MA of 188 RCTs comparing the effects of single or combination of antihypertensives with placebo or other classes of active treatments in patients with diabetes >=18 who had diabetes and CKD , of any study duration . excluded patients with no CKD	MA of 17 RCT comparing the effects of RAASI vs placebo on top of other antiHTN treatment, or vs other active treatment in patients with T2DM, with micro or macroalbuminuria >=18 years, with a follow-up >= 12 months.

Kidney failure (vs. placebo)	RAASi better than placebo*<br><br>OR 0.74 (95%CI 0.56 - 0.97)*	RAASi better than placebo *<br><br>ACE inhibitors: OR 0.68 (95%CI 0.51 – 0.91)*<br><br>ARBs: OR 0.74 (95% CI 0.57 – 0.97)*	RAASi better than placebo<br><br>ACE inhibitor: OR 0.71 (95%CI 0.39 - 1.28)<br><br>ARBs: OR 0.73 (95%CI 0.43 - 1.25)	RAASi better than placebo*<br><br>RR 0.80 (95%CI 0.69 - 0.93)	-	ARB better than placebo*<br>OR 0·81 (95%CI 0·69–0·96)<br><br>ACE inhibitor: better than placebo<br>0·73 (95%CI 0·47–1·14)	RAASi better than control (placebo and active)*<br><br>RR 0.79 (0.75 – 0.83)<br><br>ACE inhibitor: RR 0.92 (95%CI 0.84 – 0.99)*<br>ARB: RR 0.78 (95%CI 0.71–0.86)*
Kidney failure (vs. active treatment)	RAASi is not better than active treatment<br><br>0.71 (95%CI 0.40 - 1.28)	-	-	RAASi is not better than active treatment<br><br>RR 0.82 [95% CI 0.64 - 1.05]	RAASi is not better than active treatment in DM patients<br><br>DM and no DM: RR 0·87 (95%CI 0·75–0·99) *<br>DM patients only: RR 0·89 (95%CI 0·74–1·07)	-	
Doubling of SrCr (vs. placebo)	RAASi better than placebo *<br><br>OR 0.71 (95%CI 0.55 - 0.91)*	RAASi better than placebo *<br>ACE inhibitors OR 0.70 (95% CrI 0.52–0.91)*	ACE better than placebo*<br><br>ACE inhibitors: OR 0.58 (95%CI 0.32 - 0.90)*	RAASi better than placebo *<br><br>RR 0.76 (95%CI 0.69 - 0.84)*	-	-	RAASi better than control (placebo and active)<br><br>RR 0.77 (95%CI 0.50 – 1.21)<br><br>ACE inhibitor: RR 0.62 (95%CI 0.05 – 7.65)<br><br>ARB: RR 0.77 (95%CI 0.59 – 1.01)
Doubling of SrCr (vs. active treatment)	RAASi is not better than active treatment<br><br>OR 0.54 (95%CI 0.26 - 1.12)	-	-	RAASi is better than active treatment *<br><br>RR 0.66 (95%CI 0.53 - 0.83)	RAASi is not better than active treatment<br><br>DM patients only: RR 1.09 (95%CI 0.55–2.15)	-	

					DM and no DM:<br>RR 0·71<br>(95% CI 0·49–1·04)		
Regression of albuminuria (vs. placebo)	RAASi is better than placebo<br><br>OR 3.00 (95%CI 0.96 - 9.37)	-	-	RAASi better than placebo<br><br>RR 1.17 (1.00 - 1.37)	-	-	RAASi is not better than control (placebo and active)<br><br>RR 1.55 (95%CI 0.93–2.58)<br><br>ACE inhibitor: RR 1.27 (95%CI 0.98–1.71)<br><br>ARB: RR 4.10 (95%CI 0.01– >100)
Regression of albuminuria (vs. active)	RAASi is not better than active treatment<br><br>OR 1.43 (95%CI 0.91 - 2.25)	-	-	RAASi is not better than active treatment<br><br>RR 1.16 (0.99 - 1.39)	-	-	

Supp 1. Table 7: Inter-rater reliability to assess for the agreement between the two reviewers for study selection using weighted kappa statistics

Reviewer A	Reviewer B	A Yes, B Yes	A Yes, B No	A No, B Yes	A No, B No	Proportionate Agreement	Yes Probability	No Probability	Random Agreement Probability	Cohen's Kappa
**ML**	NA	87	45	72	2305	0.95337	0.00333	0.88735	0.89069	0.57341

Supp 1. Table 8: Studies' risk of bias using the Cochrane Collaboration's risk of bias scale

TRIAL/BIAS	Random sequence generation (selection bias)	Allocation concealment (selection bias)	Blinding of participants and personnel (performance bias)	Blinding of outcome assessment (detection bias)	Incomplete outcome data (attrition bias)	Selective reporting (reporting bias)	Funding
Melbourne Diabetic Nephropathy Study Group 1991	L	L	H	H	U	U	H
Chan 1992	L	U	L	L	L	L	H
Lacourciere 1993	H	U	L	U	L	L	U
Lewis 1993	U	U	L	L	L	U	U
Ravid 1993 & Ravid 1995	U	U	L	L	L	U	L
Lebovitz 1994	U	U	L	L	H	U	H
Viberti 1994	L	U	L	L	L	H	H
Agardh 1996	L	U	L	L	H	H	U
Bakris 1996	U	U	H	H	L	L	L
Schnack 1996	L	U	H	H	H	H	U
Ahmad 1997	U	U	H	H	H	L	L
Chaturvedi 1997 EUCLID Study.	L	L	L	L	L	L	H
Fogari 1997	U	U	L	L	L	U	U
Crepaldi 1998	L	L	L	U	L	L	H
Ravid 1998	L	L	L	L	L	U	L
UKPDS 1998	H	L	U	H	H	L	H
Fogari 1999	U	U	H	H	L	L	U
Muirhead 1999	U	U	L	L	L	U	H

Fogari 2000	U	U	U	U	L	L	U
HOPE 2000	L	L	L	L	L	L	H
O'Hare 2000 (The ATLANTIS Study)	L	L	U	U	L	L	H
Schrier 2000 (ABCD trial.)	U	U	L	L	H	H	U
Tarnow 2000	U	L	U	U	L	L	H
Lewis 2001 (IDNT trial)	U	L	L	L	H	H	H
Baines 2001 (ESPRIT trial)	L	L	L	L	H	H	H
Bojestig 2001	U	U	L	U	L	L	H
Brenner 2001 (RENAAL trial)	L	U	U	L	L	H	H
Parving 2001 (IRMA2 trial)	U	U	L	L	H	H	H
Kvetny 2001	U	U	L	L	H	H	H
Baba 2001 (J-MIND study)	L	L	H	H	U	U	U
Katayama 2002 (JAPAN-IDDM)	U	L	L	L	L	L	L
Fogari 2002	L	L	H	H	L	L	U
Schrier 2002	L	L	L	L	L	L	H
Ahmad 2003	U	L	L	L	U	H	U
Marre 2004 (NESTOR Trial)	U	U	L	L	L	L	H
Jerums 2004	L	L	L	H	L	L	H
Ruggenenti 2004 (The Benedict trial)	U	U	L	L	L	L	H
DallaVestra 2004	U	U	L	L	L	L	U
Fogari 2005	L	L	H	H	L	L	L

Ogawa 2007	U	U	L	H	L	U	U
Makino 2008	H	U	L	L	H	L	H
Bilous 2009	L	L	L	L	L	H	H
Mauer 2009 (RAAS trial)	L	U	L	L	L	L	H
Haller 2011 (Roadmap trial)	L	L	L	L	L	L	H
Ruggenenti 2011 (he DEMAND trial)	L	L	L	L	L	L	H
Weil 2013	L	U	L	L	L	L	H
Fuchs 2016 (PREVER-treatment trial)	L	L	L	L	L	L	L
Abbreviations: L: Low risk of bias, U: Unclear risk of bias, H: High risk of bias							

Supp 1. Table 9: Reported binary renal outcomes in the included trials (33 trials)

Trial Name	incidence of kidney failure	Incidence of doubling serum creatinine	Regression of albuminuria cases	All-cause mortality
Chan 1992				reported
Lacourciere 1993			reported	reported
Lewis 1993	reported	reported		reported
Ravid 1993			reported	
Viberti 1994				reported
Agardh 1996			reported	
Bakris 1996	reported	reported		reported

Fogari 1997			reported	
Crepaldi 1998			reported	
Ravid 1998				reported
UKPDS 1998	reported			reported
HOPE 2000	reported			reported
O'Hare 2000 (The ATLANTIS Study)			reported	
Tarnow 2000	reported			reported
Lewis 2001 (IDNT trial)	reported	reported		reported
Brenner 2001 (RENAAL trial)	reported	reported		reported
Parving 2001 (IRMA2 trial)			reported	reported
Baba 2001 (J-MIND study)			reported	
Katayama 2002 (JAPAN-IDDM)		reported		
Fogari 2002			reported	reported
Schrier 2002				reported
Marre 2004 (NESTOR Trial)			reported	reported
Jerumes 2004			reported	
Ruggenenti 2004 (The Benedict trial)				reported
DallaVestra 2004			reported	reported
Fogari 2005			reported	
Ogawa 2007			reported	
Makino 2008 (INNOVATION trial)			reported	
Bilous 2009 (DIRECT Trials)				reported

Mauer 2009 (RAAS trial)			reported	reported
Haller 2011 (Roadmap trial)	reported	reported		reported
Ruggenenti 2011 (he DEMAND trial)				reported
Weil 2013				reported

Supp 1. Table 10: Reported Continuous Renal Outcomes in the Included Trials (39 trials)

Trial Name	Serum creatinine level (umol/L)	Creatinine clearance OR GFR (ml/min)	Albuminuria level in 24 hrs sample (24hr UAE) mg/24hr	Albumin excretion rate AER or UAER (mcg/min)
Melbourne Diabetic Nephropathy Study Group 1991		reported		reported
Chan 1992	reported	reported	reported	
Lacourciere 1993	reported	reported		reported
Ravid 1993 & Ravid 1995	reported		reported	
Lebovitz 1994	reported	reported	reported	
Viberti 1994				reported
Agardh 1996	reported	reported		reported
Bakris 1996		reported	reported	
Schnack 1996		reported		

Ahmad 1997		reported		reported
Chaturvedi 1997 EUCLID Study.				reported
Fogari 1997		reported	reported	
Crepaldi 1998	reported	reported		reported
Ravid 1998		reported	reported	
Fogari 1999	reported	reported	reported	
Muirhead 1999		reported		reported
O'Hare 2000 (The ATLANTIS Study)		reported		reported
Schrier 2000 (ABCD trial.)		reported		reported
Tarnow 2000	reported	reported	reported	
Lewis 2001 (IDNT trial)	reported		reported	
Baines 2001 (ESPRIT trial)		reported		reported
Bojestig 2001		reported		reported
Brenner 2001 (RENAAL trial)	reported	reported		
Parving 2001 (IRMA2 trial)		reported		reported
Kvetny 2001		reported		
Baba 2001 (J-MIND study)	reported			reported
Katayama 2002 (JAPAN-IDDM)			reported	
Fogari 2002		reported	reported	
Schrier 2002			reported	
Ahmad 2003			reported	
Marre 2004 (NESTOR Trial)		reported		reported
Jerumes 2004		reported		reported

DallaVestra 2004				reported
Fogari 2005		reported	reported	
Bilous 2009 (DIRECT Trials)				reported
Mauer 2009 (RAAS trial)		reported		reported
Haller 2011 (Roadmap trial)		reported		
Ruggenenti 2011 (he DEMAND trial)		reported		
Evans 2012 (subgroup of Lewis 2001)		reported		

Supp 1. List 1: A list of the citations of the included clinical trials: 46 included RCT presented in 50 published articles

1. Melbourne Diabetic Nephropathy Study Group. Comparison between perindopril and nifedipine in hypertensive and normotensive diabetic patients with microalbuminuria. BMJ. 1991;302(6770):210-6.
2. Chan J, Cockram C, Nicholls M, Cheung C, Swaminathan R. Comparison of enalapril and nifedipine in treating non-insulin dependent diabetes associated with hypertension: one year analysis. BMJ. 1992;305(6860):981-5.
3. Lacourciere Y, Nadeau A, Poirier L, Tancrede G. Captopril or conventional therapy in hypertensive type II diabetics. Three-year analysis. Hypertension. 1993;21(6_pt_1):786-94.
4. Lewis EJ, Hunsicker LG, Bain RP, Rohde RD. The effect of angiotensin-converting-enzyme inhibition on diabetic nephropathy. New England Journal of Medicine. 1993;329(20):1456-62.
5. Ravid M, Savin H, Jutrin I, Bental T, Katz B, Lishner M. Long-term stabilizing effect of angiotensin-converting enzyme inhibition on plasma creatinine and on proteinuria in normotensive type II diabetic patients. Annals of Internal Medicine. 1993;118(8):577-81.
6. Lebovitz HE, Wiegmann TB, Cnaan A, Shahinfar S, Sica DA, Broadstone V, et al. Renal protective effects of enalapril in hypertensive NIDDM: role of baseline albuminuria. Kidney International Supplement 1994;45:S150-5 .
7. Viberti G, Mogensen CE, Groop LC, Pauls JF, Boner G, Van Dyk D, et al. Effect of captopril on progression to clinical proteinuria in patients with insulin-dependent diabetes mellitus and microalbuminuria. JAMA. 1994;271(4):275-9.

8. Ravid M, Neumann L, Lishner M. Plasma lipids and the progression of nephropathy in diabetes mellitus type II: effect of ACE inhibitors. Kidney international. 1995;47(3):907-10.
9. Agardh CD, Garcia-Puig J, Charbonnel B, Angelkort B, Barnett A. Greater reduction of urinary albumin excretion in hypertensive type II diabetic patients with incipient nephropathy by lisinopril than by nifedipine. Journal Of Human Hypertension. 1996;10(3):185-92.
10. Bakris GL, Copley JB, Vicknair N, Sadler R, Leurgans S. Calcium channel blockers versus other antihypertensive therapies on progression of NIDDM associated nephropathy. Kidney international. 1996;50(5):1641-50.
11. Schnack C, Hoffmann W, Hopmeier P, Schernthaner G. Renal and metabolic effects of 1-year treatment with ramipril or atenolol in NIDDM patients with microalbuminuria. Diabetologia. 1996;39(12):1611-6.
12. Ahmad J, Siddiqui MA, Ahmad H. Effective postponement of diabetic nephropathy with enalapril in normotensive type 2 diabetic patients with microalbuminuria. Diabetes Care. 1997;20(10):1576-81.
13. Chaturvedi N, Group ES. Randomised placebo-controlled trial of lisinopril in normotensive patients with insulin-dependent diabetes and normoalbuminuria or microalbuminuria. The Lancet. 1997;349(9068):1787-92.
14. Fogari R, Zoppi A, Malamani G, Lusardi P, Destro M, Corradi L. Effects of amlodipine vs enalapril on microalbuminuria in hypertensive patients with type II diabetes. Clinical Drug Investigation. 1997;13(1):42-9.
15. Crepaldi G, Carta Q, Deferrari G, Mangili R, Navalesi R, Santeusanio F, et al. Effects of lisinopril and nifedipine on the progression to overt albuminuria in IDDM patients with incipient nephropathy and normal blood pressure. Diabetes Care. 1998;21(1):104-10.
16. Ravid M, Brosh D, Levi Z, Bar-Dayan Y, Ravid D, Rachmani R. Use of enalapril to attenuate decline in renal function in normotensive, normoalbuminuric patients with type 2 diabetes mellitus: a randomized, controlled trial. Annals Of Internal Medicine. 1998;128(12 Part1):982-8.
17. UKPDS Group. Efficacy of atenolol and captopril in reducing risk of macrovascular and microvascular complications in type 2 diabetes: UKPDS 39. UK Prospective Diabetes Study Group. BMJ. 1998;317(7160):713.
18. Fogari R, Zoppi A, Corradi L, Mugellini A, Lazzari P, Preti P, et al. Long-term effects of ramipril and nitrendipine on albuminuria in hypertensive patients with type II diabetes and impaired renal function. Journal Of Human Hypertension. 1999;13(1):47-53.
19. Muirhead N, Feagan BF, Mahon J, Lewanczuk RZ, Rodger NW, Botteri F, et al. The effects of valsartan and captopril on reducing microalbuminuria in patients with type 2 diabetes mellitus: a placebo-controlled trial. Current Therapeutic Research. 1999;60(12):650-60.

20. Fogari R, Zoppi A, Corradi L, Poletti L, Pasotti M, Fogari E, et al. Long-term effects of amlodipine versus fosinopril on microalbuminuria in elderly hypertensive patients with type 2 diabetes mellitus. Current Therapeutic Research. 2000;61(3):163-73.
21. HOPE Study Investigators. Effects of ramipril on cardiovascular and microvascular outcomes in people with diabetes mellitus: results of the HOPE study and MICRO-HOPE substudy. The Lancet. 2000;355(9200):253-9.
22. O'Hare P, Bilbous R, Mitchell T, O'Callaghan C, Viberti G, Group A-ITtLAiNI-DSS. Low-dose ramipril reduces microalbuminuria in type 1 diabetic patients without hypertension: results of a randomized controlled trial. Diabetes Care. 2000;23(12):1823-9.
23. Schrier R. Effect of blood pressure control on diabetic microvascular complications in patients with hypertension and type 2 diabetes. Diab Care. 2000;23:B54-B64.
24. Tarnow L, Rossing P, Jensen C, Hansen BV, Parving HH. Long-term renoprotective effect of nisoldipine and lisinopril in type 1 diabetic patients with diabetic nephropathy. Diabetes Care. 2000;23(12):1725-30.
25. Lewis EJ, Hunsicker LG, Clarke WR, Berl T, Pohl MA, Lewis JB, et al. Renoprotective effect of the angiotensin-receptor antagonist irbesartan in patients with nephropathy due to type 2 diabetes. New England Journal of Medicine. 2001;345(12):851-60.
26. Baines L. Effect of 3 years of antihypertensive therapy on renal structure in type 1 diabetic patients with albuminuria-The European Study for the Prevention of Renal Disease in Type 1 Diabetes (ESPRIT). Diabetes. 2001;50(4):843-50.
27. Bojestig M, Karlberg BE, Lindström T, Nystrom FH. Reduction of ACE activity is insufficient to decrease microalbuminuria in normotensive patients with type 1 diabetes. Diabetes Care. 2001;24(5):919-24.
28. Brenner BM, Cooper ME, De Zeeuw D, Keane WF, Mitch WE, Parving HH, et al. Effects of losartan on renal and cardiovascular outcomes in patients with type 2 diabetes and nephropathy. New England Journal Of Medicine. 2001;345(12):861-9.
29. Parving HH, Lehnert H, Bröchner-Mortensen J, Gomis R, Andersen S, Arner P. The effect of irbesartan on the development of diabetic nephropathy in patients with type 2 diabetes. New England Journal Of Medicine. 2001;345(12):870-8.
30. Baba S, J-MIND Study Group. Nifedipine and enalapril equally reduce the progression of nephropathy in hypertensive type 2 diabetics. Diabetes research and clinical practice. 2001;54(3):191-201.
31. Katayama S, Kikkawa R, Isogai S, Sasaki N, Matsuura N, Tajima N, et al. Effect of captopril or imidapril on the progression of diabetic nephropathy in Japanese with type 1 diabetes mellitus: a randomized controlled study (JAPAN-IDDM). Diabetes Research And Clinical Practice. 2002;55(2):113-21.
32. Fogari R, Preti P, Zoppi A, Rinaldi A, Corradi L, Pasotti C, et al. Effects of amlodipine fosinopril combination on microalbuminuria in hypertensive type 2 diabetic patients. Am J Hypertens. 2002;15(12):1042–9.

33. Schrier RW, Estacio RO, Esler A, Mehler P. Effects of aggressive blood pressure control in normotensive type 2 diabetic patients on albuminuria, retinopathy and strokes. Kidney International. 2002;61(3):1086-97.
34. Ahmad J, Shafique S, Abidi SA, Parwez I. Effect of 5-year enalapril therapy on progression of microalbuminuria and glomerular structural changes in type 1 diabetic subjects. Diabetes Research And Clinical Practice. 2003;60(2):131-8.
35. Marre M, Puig JG, Kokot F, Fernandez M, Jermendy G, Opie L, et al. Equivalence of indapamide SR and enalapril on microalbuminuria reduction in hypertensive patients with type 2 diabetes: The NESTOR study. Journal Of Hypertension. 2004;22(8):1613-22.
36. Jerums G, Allen TJ, Campbell D, Cooper ME, Gilbert RE, Hammond J, et al. Long-term renoprotection by perindopril or nifedipine in non-hypertensive patients with Type 2 diabetes and microalbuminuria. Diabetic Medicine. 2004;21(11):1192-9.
37. Ruggenenti P, Fassi A, Ilieva AP, Bruno S, Iliev IP, Brusegan V, et al. Preventing Microalbuminuria in Type 2 Diabetes. N Engl J Med. 2004;351(19):1941–51.
38. Dalla Vestra M, Pozza G, Mosca A, Grazioli V, Lapolla A, Fioretto P, et al. Effect of lercanidipine compared with ramipril on albumin excretion rate in hypertensive Type 2 diabetic patients with microalbuminuria: DIAL Study (Diabete, Ipertensione, Albuminuria, Lercanidipina). Cardiovascular Therapy And Prevention. 2004;9(7):41-8.
39. Fogari R, Mugellini A, Zoppi A, Lazzari P, Destro M, Rinaldi A, et al. Effect of successful hypertension control by manidipine or lisinopril on albuminuria and left ventricular mass in diabetic hypertensive patients with microalbuminuria. European Journal Of Clinical Pharmacology. 2005;61(7):483-90.
40. Ogawa S, Takeuchi K, Mori T, Nako K, Tsubono Y, Ito S. Effects of monotherapy of temocapril or candesartan with dose increments or combination therapy with both drugs on the suppression of diabetic nephropathy. Hypertension Research. 2007;30(4):325-34.
41. Makino H, Haneda M, Babazono T, Moriya T, Ito S, Iwamoto Y, et al. Microalbuminuria reduction with telmisartan in normotensive and hypertensive Japanese patients with type 2 diabetes: a post-hoc analysis of The Incipient to Overt: Angiotensin II Blocker, Telmisartan, Investigation on Type 2 Diabetic Nephropathy (INNOVATION) study. Hypertension Research. 2008;31(4):657-64.
42. Bilous R, Chaturvedi N, Sjølie AK, Fuller J, Klein R, Orchard T, et al. Effect of candesartan on microalbuminuria and albumin excretion rate in diabetes: three randomized trials. Annals Of Internal Medicine. 2009;151(1):11-20.
43. Chaturvedi N, Porta M, Klein R, Orchard T, Fuller J, Parving HH, et al. Effect of candesartan on prevention (DIRECT-Prevent 1) and progression (DIRECT-Protect 1) of retinopathy in type 1 diabetes: randomised, placebo-controlled trials. The Lancet. 2008;372(9647):1394-402.

44. Sjølie AK, Klein R, Porta M, Orchard T, Fuller J, Parving HH, et al. Effect of candesartan on progression and regression of retinopathy in type 2 diabetes (DIRECT-Protect 2): a randomised placebo-controlled trial. The Lancet. 2008;372(9647):1385-93.
45. Mauer M, Zinman B, Gardiner R, Suissa S, Sinaiko A, Strand T, et al. Renal and retinal effects of enalapril and losartan in type 1 diabetes. New England Journal Of Medicine. 2009;361(1):40-51.
46. Haller H, Ito S, Izzo Jr JL, Januszewicz A, Katayama S, Menne J, et al. Olmesartan for the delay or prevention of microalbuminuria in type 2 diabetes. New England Journal of Medicine. 2011;364(10):907-17.
47. Ruggenenti P, Fassi A, Ilieva AP, Iliev IP, Chiurchiu C, Rubis N, et al. Effects of verapamil added-on trandolapril therapy in hypertensive type 2 diabetes patients with microalbuminuria: the BENEDICT-B randomized trial. Journal of hypertension. 2011;29(2):207-16.
48. Evans M, Bain SC, Hogan S, Bilous RW. Irbesartan delays progression of nephropathy as measured by estimated glomerular filtration rate: post hoc analysis of the Irbesartan Diabetic Nephropathy Trial. Nephrology Dialysis Transplantation. 2012;27(6):2255-63.
49. Weil EJ, Fufaa G, Jones LI, Lovato T, Lemley KV, Hanson RL, et al. Effect of losartan on prevention and progression of early diabetic nephropathy in American Indians with type 2 diabetes. Diabetes. 2013;62(9):3224-31.
50. Fuchs FD, Scala LCN, Vilela-Martin JF, de Mello RB, Mosele F, Whelton PK, et al. Effectiveness of chlorthalidone/amiloride versus losartan in patients with stage I hypertension: results from the PREVER-treatment randomized trial. Journal Of Hypertension. 2016;34(4):798-806.
