# Supplementary material for: The effect of renin–angiotensin–aldosterone system inhibitors on continuous and binary kidney outcomes in subgroups of patients with diabetes: a meta-analysis of randomized clinical trials
Source: BMC Nephrol. 2022 Apr 28;23:161. doi: 10.1186/s12882-022-02763-1 (PMC9052620; doi:10.1186/s12882-022-02763-1)
Supplement: Supplementary file 2 — Additional file 2: Supplementary 2. [file 12882_2022_2763_MOESM2_ESM.pdf]

The Effect of Renin-Angiotensin-Aldosterone System Inhibitors on Binary and Continuous Renal Outcomes in Subgroups of Patients with Diabetes: An Extensive Meta-Analysis

Supplement 2

Table of Contents

SUPP 2. TABLE 1: SUBGROUP ANALYSIS OF MEAN DIFFERENCE OF EGFR IN RAASI *VERSUS* PLACEBO 2

SUPP 2. TABLE 2: SUBGROUP ANALYSIS OF MEAN DIFFERENCE OF EGFR IN RAASI *VERSUS* ACTIVE TREATMENTS. 3

SUPP 2. TABLE 3: SUBGROUP ANALYSIS OF SERUM CREATININE LEVELS IN RAASI *VERSUS* PLACEBO.................... 4

SUPP 2. TABLE 4: SUBGROUP ANALYSIS OF SERUM CREATININE LEVELS IN RAASI *VERSUS* ACTIVE TREATMENTS . 5

SUPP 2. TABLE 5: SUBGROUP ANALYSIS OF ALBUMINURIA LEVEL IN RAASI *VERSUS* PLACEBO 7

SUPP 2. TABLE 6: SUBGROUP ANALYSIS OF ALBUMINURIA LEVEL IN RAASI *VERSUS* ACTIVE TREATMENTS 8

SUPP 2. FIGURE 1A: FOREST PLOT FOR KIDNEY FAILURE IN TRIALS COMPARING RAAS INHIBITORS *VERSUS* PLACEBO 10

SUPP 2. FIGURE 2A: FOREST PLOT FOR DOUBLING OF SERUM CREATININE IN TRIALS COMPARING RAAS INHIBITORS *VERSUS* PLACEBO 10

SUPP 2. FIGURE 2B: FOREST PLOT FOR DOUBLING OF SERUM CREATININE IN TRIALS COMPARING RAAS INHIBITORS *VERSUS* OTHER ANTI-HYPERTENSIVES 11

SUPP 2. TABLE 7: META ANALYSIS FOR BINARY OUTCOMES OF RAASI *VERSUS* PLACEBO................................. 13

SUPP 2. TABLE 8: META ANALYSIS FOR BINARY OUTCOMES OF RAASI VERSUS ACTIVE TREATMENTS 13

SUPP 2. TABLE 9: SUBGROUP ANALYSIS FOR KIDNEY FAILURE RAASI *VERSUS* PLACEBO................................... 14

SUPP 2. TABLE 10: SUBGROUP ANALYSIS FOR KIDNEY FAILURE RAASI *VERSUS* ACTIVE TREATMENTS................ 15

SUPP 2. TABLE 11: SUBGROUP ANALYSIS FOR DOUBLING OF SRCR IN RAASI *VERSUS* PLACEBO 16

SUPP 2. TABLE 12: SUBGROUP ANALYSIS FOR DOUBLING OF SRCR IN RAASI *VERSUS* ACTIVE TREATMENTS 17

SUPP 2. TABLE 13: SUBGROUP ANALYSIS FOR REGRESSION OF ALBUMINURIA IN RAASI *VERSUS* PLACEBO 18

SUPP 2. TABLE 14: SUBGROUP ANALYSIS FOR REGRESSION OF ALBUMINURIA IN RAASI *VERSUS* ACTIVE TREATMENT.. 19

SUPP 2. TABLE 15: SUBGROUP ANALYSIS FOR ALL-CAUSE MORTALITY IN RAASI *VERSUS* PLACEBO 20

SUPP 2. TABLE 16: SUBGROUP ANALYSIS FOR ALL-CAUSE MORTALITY IN RAASI *VERSUS* ACTIVE TREATMENTS .. 21

SUPP 2. TABLE 17: SUBGROUP ANALYSIS FOR DISRUPTIVE COUGH IN RAASI *VERSUS* PLACEBO 23

SUPP 2. TABLE 18: SUBGROUP ANALYSIS FOR DISRUPTIVE COUGH IN RAASI *VERSUS* ACTIVE TREATMENT......... 24

SUPP 2. TABLE 19: SUBGROUP ANALYSIS FOR THE NEED OF ADDITIONAL ANTIHYPERTENSIVES IN RAASI *VERSUS* PLACEBO 25

SUPP 2. TABLE 20: SUBGROUP ANALYSIS FOR THE NEED OF ADDITIONAL ANTIHYPERTENSIVES IN RAASI *VERSUS* ACTIVE TREATMENT 25

SUPP 2. TABLE 21: META-ANALYSIS OF WITHDRAWAL REASONS IN RAASI *VERSUS* PLACEBO 27

SUPP 2. TABLE 22: META-ANALYSIS OF WITHDRAWAL REASONS IN RAASI *VERSUS* ACTIVE TREATMENTS 27

SUPP 2. TABLE 23: RESULTS FOR OVERALL META- ANALYSIS FOR RAASI VERSUS PLACEBO OR OTHER ANTI-HTN DRUGS (CONTINUOUS OUTCOMES) 27

SUPP 2. TABLE 24A: SENSITIVITY ANALYSIS RESULTS FOR OVERALL META- ANALYSIS AFTER EXCLUDING OUTLIER STUDIES........ 28

SUPP 2. TABLE 25: EXCLUDED STUDIES FROM SENSITIVITY ANALYSIS 29

Supp 2. Table 1: Subgroup analysis of mean difference of eGFR in RAASi *versus* placebo

Subgroup Analysis According to :	No. Of Trials	N° patients RAASi /Placebo	RMD	95% CI	I²	*p* value *
**Type of diabetes**						
Type 1 DM	5	302/211	-0.9	-7.8; 6.01	7%	0.36
Type 2 DM	7	2785/2749	-0.49	-7.26; 6.28	92%	<0.01
**Albuminuria**						
Microalbuminuria	7	423/408	2.2	-4.26; 8.65	69%	<0.01
Normoalbuminuria	3	2519/2407	-0.12	-5.77; 5.53	95%	<0.01
Missing	1	18/18	-7.2	-19.58; 5.18	NA	
Mixed	1	127/127	-13.8	-18.87, -8.73	NA	
**CKD status**						
CKD any stage	2	2255/2242	4.64	-13.20; 22.48	91%	<0.01
No CKD	9	814/700	-2.64	-7.19; 1.91	86%	<0.01
Missing	1	18/18	10	-3.96; 23.96	NA	
**HTN status**						
Yes	8	2613/2574	-5.86	-12.92; 1.20	81%	<0.01
No	4	474/386	2.22	-3.36; 7.79	57%	0.02
**DM duration**						
< 10 years	6	2725/2718	-1.04	-8.51; 6.44	93%	<0.01
≥ 10 years	5	302/211	-0.9	-7.80; 6.01	7%	0.36
Missing	1	60/31	6.76	-11.13; 24.64	NA	
**Age categories**						
< 60 years	11	2960/2833	0.55	-3.81; 4.90	83%	<0.01
≥ 60 years	1	127/127	-13.8	-18.87; -8.73	NA	
**BMI categories**						
Missing	3	59/63	6.01	-7.19; 19.20	72%	0.03

< 30	6	542/450	-2.29	-8.28; 3.70	89%	<0.01
≥ 30	3	2486/2447	-3.65	-8.90; 1.60	7%	0.34
**Study size**						
< 100	5	151/128	4.81	-3.29; 12.92	51%	0.09
≥ 100	7	2936/2832	-3.39	-8.11; 1.34	90%	<0.01
**Study duration**						
≤ 2years	4	316/296	-0.08	-9.60; 9.45	51%	0.1
> 2years	8	2771/2664	-1.02	-6.77; 4.73	90%	<0.01
**Year of Publication**						
< 2000	4	241/213	3.46	-0.06; 6.97	12%	0.33
≥ 2000	8	2846/2747	-2.54	-9.25; 4.16	77%	<0.01

Supp 2. Table 2: Subgroup analysis of mean difference of eGFR in RAASi *versus* active treatments

Subgroup Analysis According to :	No. Of Trials	N° patients RAASi /Placebo	RMD	95% CI	I^2	*p* value *
**Type of diabetes**						
Type 1 DM	3	75/71	-5.38	-10.66; -0.11	2%	0.36
Type 2 DM	12	1136/1164	-0.4	-4.48; 3.68	88%	<0.01
Both	1	23/27	-1	-9.70;7.70	NA	
**Albuminuria**						
Microalbuminuria	8	598/596	-0.72	-6.73; 5.29	71%	<0.01
Mixed	3	286/299	-0.28	-3.14; 2.58	3%	0.36
Macroalbuminuria	4	332/349	-0.37	-6.12; 5.39	95%	<0.01
Missing	1	18/18	-9.31	-22.24; 3.62	NA	
**CKD status**						
CKD any stage	7	573/595	-0.76	-4.53; 3.00	91%	<0.01
No CKD	9	661/667	-1.2	-6.66; 4.27	68%	<0.01
**HTN status**						
Missing	1	23/27	-1	-9.70; 7.70	NA	
Yes	12	1138/1164	-0.6	-4.63; 3.44	90%	<0.01
No	3	73/71	-4.53	-11.58; 2.53	0%	0.44
**DM duration**						
Missing	1	46/45	21	9.11; 32.89	NA	
< 10 years	10	1072/1085	-1.92	-3.96; 0.13	47%	0.05
≥ 10 years	5	116/132	-1.01	-6.82; 4.79	93%	<0.01
**Age categories**						
< 60 years	13	928/955	-1.3	-3.83; 1.23	85%	<0.01

≥ 60 years	3	306/307	3.6	-12.36; 19.57	90%	<0.01
**BMI categories**						
Missing	4	232/239	-4.43	-10.18; 1.33	0%	0.71
< 30	9	695/701	-1.07	-6.21; 4.08	80%	<0.01
≥ 30	3	307/322	1.97	-3.70; 7.63	92%	<0.01
**Study size**						
< 100	10	332/362	-1.29	-6.83; 4.26	90%	<0.01
≥ 100	6	902/900	-0.65	-1.86; 0.57	18%	0.3
**Study duration**						
≤ 2years	8	713/712	-0.16	-5.88; 5.56	71%	<0.01
> 2years	8	521/550	-1.8	-5.55; 1.96	92%	<0.01
**Year of Publication**						
< 2000	9	484/509	0.61	-4.84; 6.05	88%	<0.01
≥ 2000	7	750/753	-2.7	-5.14; -0.26	51%	0.06

Supp 2. Table 3: Subgroup analysis of serum creatinine levels in RAASi *versus* placebo

Subgroup Analysis According to :	No. Of Trials	N° patients RAASi /Placebo	MD	95% CI	I^2	*p* value *
**Type of diabetes**						
Type1DM	1	32/34	-16	-24.36; -7.64		
Type2DM	3	691/672	-12.91	-16.46; -9.35	0%	0.99
**Albuminuria**						
Micro	2	81/79	-13.59	-17.36; -9.81	0%	0.52
Mixed	1	63/58	-11.62	-42.41; 19.16		
Macro	1	579/569	-12.42	-22.21; -2.63		
**CKD status**						
CKD any stage	2	642/627	-12.35	-21.67; -3.02	0%	0.96
No CKD	2	81/79	-13.59	-17.36; -9.81	0%	0.52
**HTN status**						
Yes	2	642/627	-12.35	-21.67; -3.02	0%	0.96
No	2	81/79	-13.59	-17.36; -9.81	0%	0.52

**DM duration**						
<10 years	2	112/103	- 12.98	-16.79; - 9.17	0%	0.93
>= 10 years	1	32/34	-16	-24.36; - 7.64		
missing	1	579/569	- 12.42	-22.21; - 2.63		
**Age categories**						
<60 years	4	723/706	-13.4	-16.78; - 10.01	0%	0.93
>= 60 years	0					
**BMI categories**						
missing	2	112/103	- 12.98	-16.79; - 9.17	0%	0.93
<30	1	32/34	-16	-24.36; - 7.64		
>=30	1	579/569	- 12.42	-22.21; - 2.63		
**Study size**						
<100	2	81/79	- 13.59	-17.36; - 9.81	0%	0.52
>=100	2	642/627	- 12.35	-21.67; - 3.02	0%	0.96
**Study duration**						
<=2years	0					
> 2years	4	723/706	-13.4	-16.78; - 10.01	0%	0.93
**Year of Publication**						
<2000	3	144/137	- 13.54	-17.21; - 9.87	0%	0.81
>=2000	1	579/569	- 12.42	-22.21; - 2.63		

Supp 2. Table 4: Subgroup analysis of serum creatinine levels in RAASi *versus* active treatments

Subgroup Analysis According to :	No. Of Trials	N° patients RAASi /Other Anti-HTN	MD	95% CI	I^2	*p* value
**Type of diabetes**						
Type1DM	2	57/53	-8.78	-23.2; 5.64	15%	0.28
Type2DM	6	1093/1107	2.04	-4.04; 8.12	67%	0.01
**Albuminuria**						

Micro	1	32/26	-12	-19.12; -4.88		
Mixed	4	460/487	3.54	-1.17; 8.25	58%	0.07
Macro	3	658/647	-1.8	-14.9; 11.31	54%	0.12
**CKD status**						
CKD any stage	6	1084/1094	2.27	-4.28; 8.82	67%	0.01
No CKD	2	66/66	-5.5	-18.14; 7.15	82%	0.02
**HTN status**						
Yes	7	1118/1134	2.11	-3.43; 7.64	60%	0.02
No	1	32/26	-12	-19.12; -4.88		
**DM duration**						
<10 years	5	514/540	3.82	-0.52; 8.15	47%	0.11
>= 10 years	2	57/53	-8.78	-23.2; 5.64	15%	0.28
missing	1	579/567	-10.62	-20.40; -0.84		
**Age categories**						
<60 years	6	917/905	-1.49	-8.91; 5.94	71%	<0.01
>= 60 years	2	233/255	6.28	2.52; 10.03	0%	0.93
**BMI categories**						
missing	2	376/395	2.93	-3.54; 9.4	82%	0.02
<30	4	141/145	-0.47	-10.58; 9.63	75%	<0.01
>=30	2	633/620	-2.26	-20.31; 15.8	74%	0.05
**Study size**						
<100	5	195/198	0.87	-7.96; 9.7	72%	<0.01
>=100	3	955/962	-0.77	-9.9; 8.35	76%	<0.01
**Study duration**						
<=2years	4	480/500	4.38	-0.66; 9.42	59%	0.06
> 2years	4	670/660	-6.36	-14.46; 1.75	56%	0.08
**Year of Publication**						
<2000	5	338/338	0.24	-7.59; 8.07	72%	<0.01

>=2000	3	812/822	-0.3	-11.51; 10.91	80%	<0.01

Supp 2. Table 5: Subgroup analysis of albuminuria level in RAASi *versus* placebo

Subgroup Analysis According to :	No. Of Trials	N° patients RAASi /Placebo	SMD	C95% CI	I²	*p* value *
**Type of diabetes**						
Type 1 DM	8	437/320	-1.05	-2.00; -0.10	66%	<0.01
Type 2 DM	6	475/452	-1.06	-1.77; -0.36	90%	<0.01
Both	1	2613/2618	-0.37	-0.42; -0.32	NA	
**Albuminuria**						
Microalbuminuria	10	555/535	-1.3	-2.07; -0.53	94%	<0.01
Normoalbuminuria	3	2900/2810	-0.44	-1.30; -0.42	97%	<0.01
Missing	2	70/45	-0.42	-0.80; -0.04	0%	0.87
**CKD status**						
CKD any stage	1	23/27	0.62	0.05; 1.19	NA	
No CKD	13	3484/3345	-1.2	-1.77, -064	96%	<0.01
Missing	1	18/18	0.1	-0.55; 0.75	NA	
**HTN status**						
Yes	4	2919/2877	-0.76	-1.19; -0.33	96%	<0.01
No	11	606/513	-1.1	-1.86; -0.33	96%	<0.01
**DM duration**						
<10 years	6	452/457	-1.3	-2.12; -0.47	92%	<0.01
≥ 10 years	9	3013/2902	-0.8	-1.67; -0.08	95%	<0.01
**Age categories**						
<60 years	14	3488/3354	-0.91	-1.47; -0.34	96%	<0.01
≥ 60 years	1	37/36	-2.4	-3.01; -1.79	NA	
**BMI categories**						

Missing	6	206/181	-0.61	-1.40; 0.18	92%	<0.01
< 30	7	3065/2977	-1.32	-2.31; -0.32	97%	<0.01
≥ 30	2	254/232	-1.16	-1.51; -0.82	52%	<0.01
**Study size**						
<100	9	335/282	-1.14	-1.98; -0.30	94%	<0.01
≥ 100	6	3525/3390	-0.81	-1.50; -0.11	97%	<0.01
**Study duration**						
≤ 2years	6	414/369	-0.82	-2.00; -0.28	97%	<0.01
> 2years	9	3111/3021	-1.14	-1.46; -0.18	91%	<0.01
**Year of Publication**						
<2000	6	336/304	-1.83	-2.53; -1.12	89%	<0.01
≥ 2000	9	3189/3086	-0.45	-1.03, 0.14	95%	<0.01
**Mean type**						
Arithmetic	9	3289/3181	-0.86	-1.59; -0.14	96%	<0.01
Geometric	6	236/209	-1.22	-2.17; -0.27	94%	<0.01

Supp 2. Table 6: Subgroup analysis of albuminuria level in RAASi *versus* active treatments

**Subgroup Analysis According to :**	**No. Of Trials**	**N° patients RAASi /Placebo**	**SMD**	**C95% CI**	**I^2**	***p* value ***
**Type of diabetes**						
Type 1 DM	3	75/71	-1.47	-3.10; 0.16	93%	<0.01
Type 2 DM	14	1573/1614	-0.4	-0.74; -0.05	89%	<0.01
Both	1	23/27	-0.26	-0.82; 0.29	NA	
**Albuminuria**						
Macroalbuminuria	4	332/349	-1.55	-3.06; -0.04	97%	<0.01
Microalbuminuria	8	641/642	-0.26	-0.44; -0.08	10%	0.35
Mixed	5	680/703	-0.28	-0.60; 0.04	90%	<0.01

Missing	1	18/18	-0.41	-1.08; 0.25	NA	
**CKD status**						
CKD any stage	9	967/999	-0.79	-1.57; -0.02	95%	<0.01
No CKD	9	704/713	-0.31	-0.49; -0.13	34%	0.15
**HTN status**						
Yes	14	1329/1380	-0.57	-1.12; -0.03	92%	<0.01
No	4	3019/305	-0.63	-0.90; -0.37	0%	0.44
**DM duration**						
< 10 years	12	1466/1489	-0.44	-0.84; -0.04	91%	<0.01
≥ 10 years	6	205/223	-0.81	-1.72, 0.11	90%	<0.01
**Age categories**						
< 60 years	11	732/736	-0.83	-1.44; -0.22	92%	<0.01
≥ 60 years	7	939/976	-0.15	-0.28; -0.01	43%	0.11
**BMI categories**						
Missing	5	380/409	-0.12	-0.34; 0.10	45%	0.12
< 30	9	738/747	-0.58	-1.20; 0.03	86%	<0.01
≥ 30	4	553/556	-0.95	-2.05; 0.16	96%	<0.01
**Study size**						
< 100	9	286/317	-0.95	-1.67; -0.22	92%	<0.01
≥ 100	9	1385/1395	-0.21	-0.37,-0.05	79%	<0.01
**Study duration**						
≤ 2years	9	904/928	-0.39	-0.93; 016	92%	<0.01
> 2years	9	767/784	-0.73	-1.30; -0.15	86%	<0.01
**Year of Publication**						
< 2000	8	438/464	-0.62	-1.21; -0.03	92%	<0.01
≥ 2000	10	1233/1248	-0.50	-1.06;0.05	89%	<0.01
**Mean type**						
Arithmetic	9	697/729	-0.49	-1.04; 0.06	92%	<0.01

Geometric	9	974/983	-0.62	-1.22; -0.03	88%	<0.01

Supp 2. Figure 1A: Forest plot for kidney failure in trials comparing RAAS inhibitors *versus* placebo

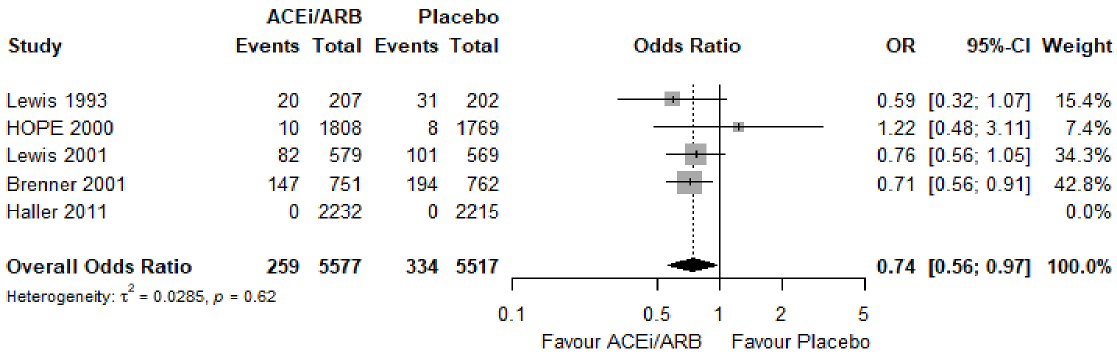

Supp 2. Figure 1B: Forest plot for kidney failure in trials comparing RAAS inhibitors *versus* other anti-hypertensives

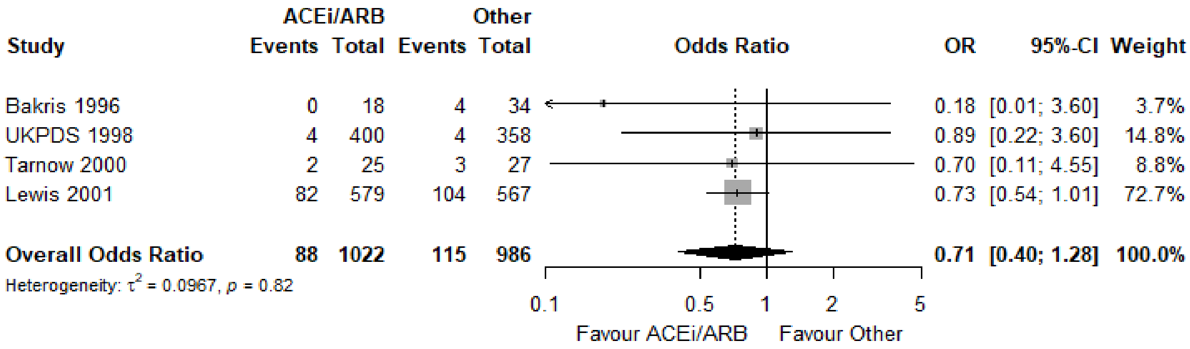

Supp 2. Figure 2A: Forest plot for doubling of serum creatinine in trials comparing RAAS inhibitors *versus* placebo

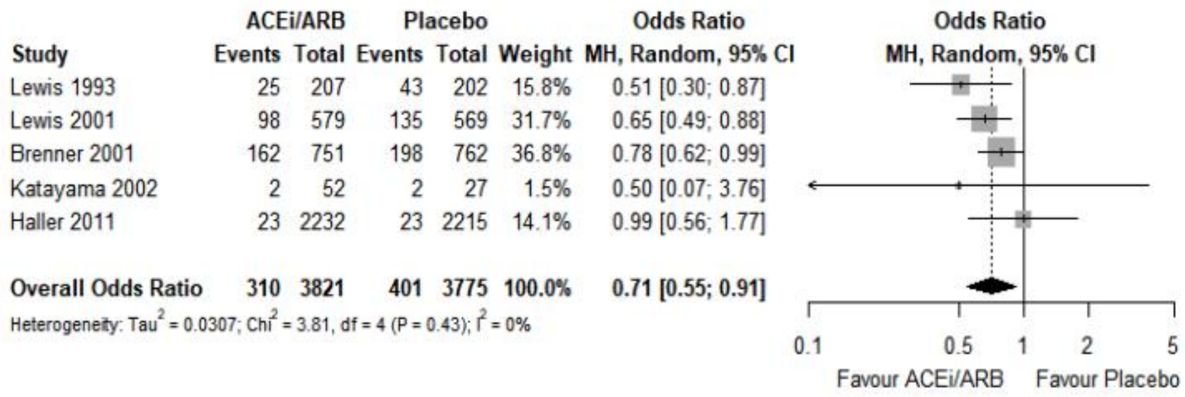

Supp 2. Figure 2B: Forest plot for doubling of serum creatinine in trials comparing RAAS inhibitors *versus* other anti-hypertensives

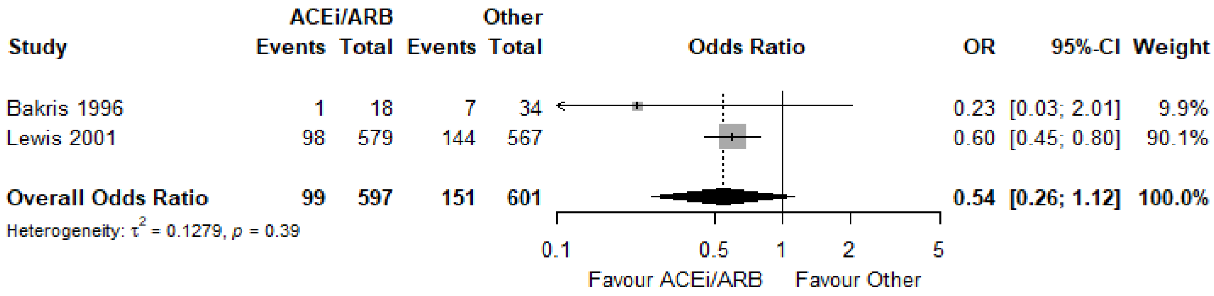

Supp 2. Figure 3A: Forest plot for regression of albuminuria in trials comparing RAAS inhibitors *versus* placebo

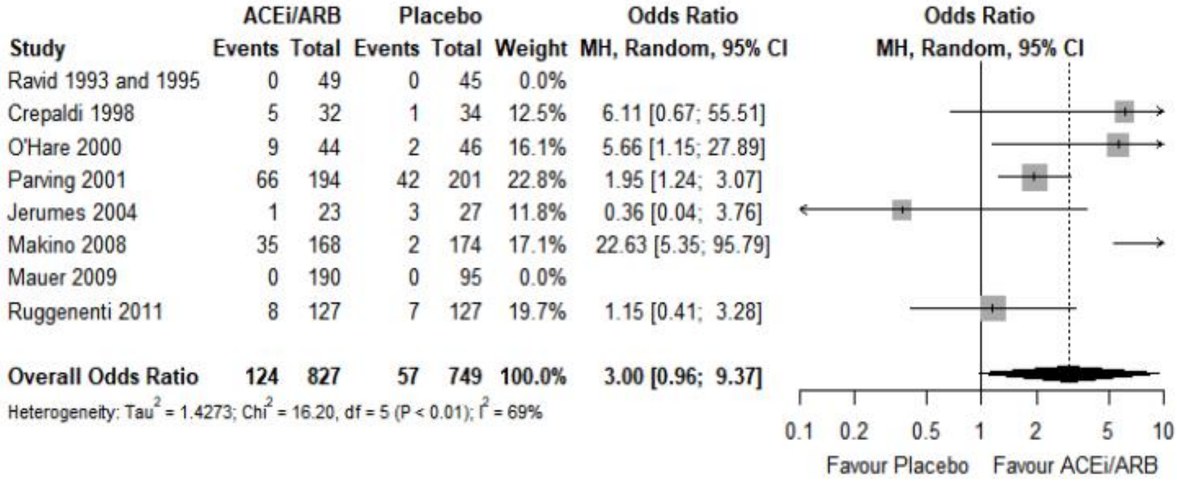

Supp 2. Figure 3B: Forest plot for regression of albuminuria in trials comparing RAAS inhibitors *versus* other anti-hypertensives

Study	ACEi/ARB Events	ACEi/ARB Total	Other Events	Other Total	Weight	Odds Ratio MH, Random, 95% CI
Agardh 1996	43	168	22	167	14.4%	2.27 [1.29; 4.00]
Fogari 1997	4	25	3	25	5.5%	1.40 [0.28; 7.00]
Crepaldi 1998	5	32	0	26	2.1%	10.60 [0.56; 201.27]
Baba 2001	11	208	19	228	12.1%	0.61 [0.29; 1.32]
Fogari 2002	47	102	34	103	14.4%	1.73 [0.98; 3.05]
Marre 2004	120	286	112	283	16.9%	1.10 [0.79; 1.54]
Jerums 2004	1	23	3	27	3.1%	0.36 [0.04; 3.76]
DallaVestra 2004	28	89	21	91	13.3%	1.53 [0.79; 2.97]
Fogari 2005	27	61	19	60	12.4%	1.71 [0.82; 3.60]
Ogawa 2007	16	74	2	18	5.7%	2.21 [0.46; 10.62]
**Overall Odds Ratio**	**302**	**1068**	**235**	**1028**	**100.0%**	**1.43 [0.91; 2.25]**

Heterogeneity: $Tau^2 = 0.2872$; $Chi^2 = 13.40$, df = 9 (P = 0.15); $I^2 = 33\%$

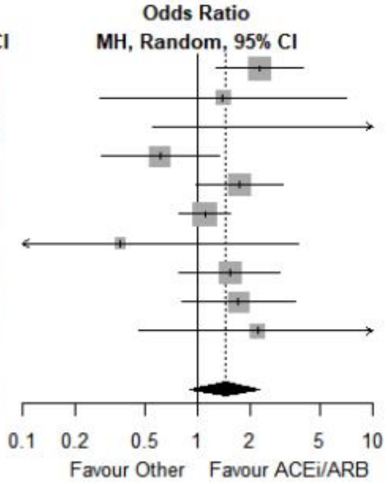

Supp 2. Figure 4A: Forest plot for all-cause mortality in trials comparing RAAS inhibitors *versus* placebo

Study	ACEi/ARB Events	ACEi/ARB Total	Placebo Events	Placebo Total	Weight	Odds Ratio MH, Random, 95% CI
Lewis 1993	8	207	14	202	8.3%	0.54 [0.22; 1.32]
Viberti 1994	0	46	1	46	1.0%	0.33 [0.01; 8.22]
Ravid 1998	3	97	2	97	2.9%	1.52 [0.25; 9.28]
HOPE 2000	196	1808	248	1769	19.0%	0.75 [0.61; 0.91]
Lewis 2001	87	579	93	569	17.2%	0.91 [0.66; 1.24]
Brenner 2001	158	751	155	762	18.3%	1.04 [0.81; 1.34]
Parving 2001	3	194	1	201	2.0%	3.14 [0.32; 30.46]
Bilous 2009	51	2613	48	2618	15.8%	1.07 [0.72; 1.59]
Mauer 2009	2	190	1	95	1.8%	1.00 [0.09; 11.17]
Haller 2011	26	2232	15	2215	11.7%	1.73 [0.91; 3.27]
Ruggenenti 2011	1	127	3	127	1.9%	0.33 [0.03; 3.20]
**Overall Odds Ratio**	**535**	**8844**	**581**	**8701**	**100.0%**	**0.96 [0.69; 1.34]**

Heterogeneity: $Tau^2 = 0.1422$; $Chi^2 = 13.35$, df = 10 (P = 0.20); $I^2 = 25\%$

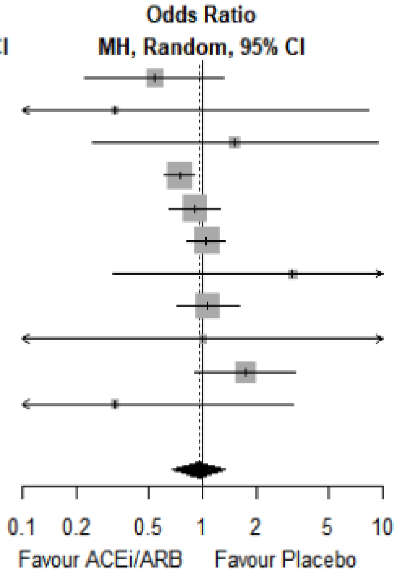

Supp 2. Figure 4B: Forest plot for all-cause mortality in trials comparing RAAS inhibitors *versus* other anti-hypertensives

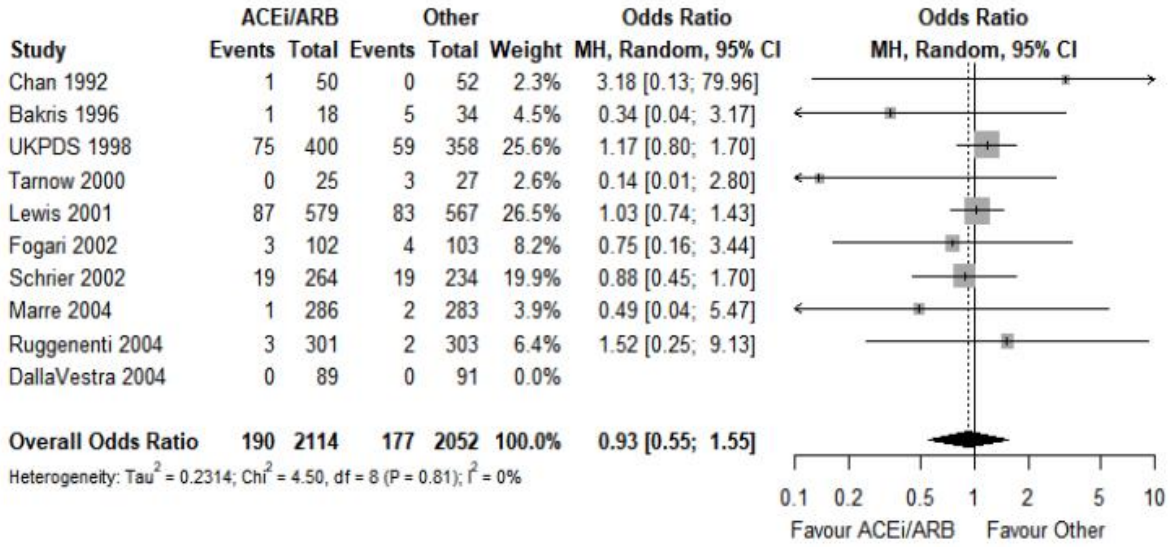

Supp 2. Table 7: Meta analysis for binary outcomes of RAASi *versus* placebo

Table. Meta analysis for binary outcomes					
**Outcome**	**No. Of Trials**	**Pooled cumulative incidence rate**		**Heterogeneity I^2**	**Odds ratio (95% CI)**
		**RAASi**	**Placebo**		
**Kidney failure**	5	259/5577	334/5517	0%	0.74 (0.56; 0.97)*
**Doubling of SrCr**	5	310/3821	401/3775	0% (0.43)	0.71 (0.55; 0.91)*
**All-cause mortality**	11	535/8844	581/8701	25%(0.20)	0.96 (0.69; 1.34)
**Regression of Albuminuria**	8	124/827	57/749	69% (<0.01)	3.00(0.96; 9.37)
**Require Anti-HTN**	10	472/3191	675/3203	34% (0.13)	0.39 (0.23;0.67)*
**Disruptive Cough**	5	52/613	14/488	3% (0.39)	2.67 (0.80; 8.89)

Supp 2. Table 8: Meta analysis for binary outcomes of RAASi versus active treatments

**Outcome**	**No. Of Trials**	**Pooled cumulative incidence rate**		**Heterogeneity I^2**	**Odds ratio (95% CI)**
		**RAASi**	**Other Anti-HTN**		
**Kidney failure**	4	88/1022	115/986	0%(0.82)	0.71 (0.40;1.28

**Doubling of SrCr**	2	99/597	151/601	0% (0.39)	0.54 (0.26; 1.12)
**All-cause mortality**	10	190/2114	177/2052	0% (0.81)	0.93 (0.55; 1.55)
**Regression of Albuminuria**	10	302/1068	235/1028	33% (0.15)	1.43 (0.91;2.25)
**Require Anti-HTN**	10	351/978	326/1018	58% (0.01)	1.38 (0.84; 2.29)
**Disruptive Cough**	9	97/1376	19/1431	7% (0.38)	6.57 (2.67;16.16)*

Supp 2. Table 9: Subgroup Analysis for kidney failure RAASi *versus* Placebo Table.

**Subgroup Analysis According to :**	**No. Of Trials**	**Pooled cumulative incidence rate**		**Heterogeneity I^2**	**Odds ratio (95% CI)**
		**RAASi**	**Placebo**		
**Type of diabetes**					
Type 1 DM	1	20/207	31/202	NA	0.59 (0.32; 1.07)
Type 2 DM	3	229/3562	295/3546	0%(*p*=0.73)	0.73 (0.60; 0.89)*
Both	1	10/1808	8/1769	NA	1.22 (0.48; 3.11)
**Albuminuria**					
Normoalbuminuria	1	0/2232	0/2215	NA	NA
Mixed	1	10/1808	8/1769	NA	1.22 (0.48; 3.11)
Macroalbuminuria	2	229/1330	295/1331	0% (*p*=0.73)	0.73 (0.60; 0.89)*
Missing	1	20/207	31/202	NA	0.59 (0.32; 1.07)
**CKD status**					
CKD any stage	4	249/3769	326/3748	0%(0.75)	0.72 (0.59; 0.87)*
No CKD	1	10/1808	8/1769	NA	1.22 (0.48; 3.11)
**HTN status**					
Yes	5	259/5577	334/5517	0% (0.62)	0.74 (0.56; 0.97)*
No	0				
**DM duration**					
<10 years	3	229/3562	295/3546	40%(0.20)	0.78 (0.38; 1.59)
≥ 10 years	2	30/2015	39/1971	0%(*p*=0.73)	0.73 (0.60; 0.89)*
**Age categories**					

<60 years	3	102/3018	132/2986	0% (0.45)	0.72 (0.52; 0.98)*
≥ 60 years	2	157/2559	202/2531	18%(0.62)	0.79 (0.50; 1.27)
**BMI categories**					
Missing	1	20/207	31/202	NA	0.59 (0.32; 1.07)
<30	2	157/2559	202/2531	18%(0.62)	0.79 (0.50; 1.27)
≥30	2	82/2811	101/2784	NA	0.76 (0.56; 1.05)
**Study size**					
<100	0				
≥100	5	259/5577	334/5517	0	0.74 (0.56; 0.97)
**Study duration**					
≤ 2years					
> 2years	5	259/5577	334/5517	0% (0.62)	0.74 (0.56; 0.97)*
**Year of Publication**					
<2000	1	20/207	31/202	NA	0.59 (0.32; 1.07)
≥2000	4	239/5370	303/5315	0%	0.77 (0.58; 1.01)

Supp 2. Table 10: Subgroup Analysis for kidney failure RAASi *versus* active treatments

Subgroup Analysis According to :	No. Of Trials	Pooled cumulative incidence rate		Heterogeneity I^2	Odds ratio (95% CI)
		RAASi	Other Anti-HTN		
**Type of diabetes**					
Type 1 DM	1	2/25	3/27	NA	0.70 (0.40; 1.28)
Type 2 DM	3	86/997	112/959	0% (0.63)	0.71 (0.33; 1.49)
**Albuminuria**					
Macroalbuminuria	3	84/622	111/628	0% (0.66)	0.68 (0.34; 1.37)
Mixed	1	4/400	4/358	NA	0.89 (0.22; 3.60)
**CKD status**					
CKD any stage	3	84/622	111/628	0% (0.66)	0.68 (0.34; 1.37)
No CKD	1	4/400	4/358	NA	0.89 (0.22; 3.60)
**HTN status**					

Yes	4	88/1022	115/986	0% (0.82)	0.71 (0.40; 1.28)
No	0				
**DM duration**					
<10 years	1	4/400	4/358	NA	0.89 (0.22; 3.60)
≥ 10 years	2	2/43	7/61	0% (0.55)	0.46 (0.08;2.57)
Missing	1	82/579	104/567	0% (0.82)	0.73 (0.54;1.01)
**Age categories**					
<60 years	3	88/1004	111/952	0% (0.96)	0.74 (0.55; 1.01)
≥ 60 years	1	0/18	4/34	NA	0.18 (0.01;3.6)
**BMI categories**					
<30	2	82/597	108/601	0% (0.36)	0.63 (0.23; 1.77)
≥ 30	2	6/425	7/385	0%(0.83)	0.82 (0.27; 2.51)
**Study size**					
<100	2	2/43	7/61	0%(0.46)	0.46 (0.08; 2.57)
≥ 100	2	86/979	108/925	0%(0.79)	0.74 (0.54;101)
**Study duration**					
≤2years	0				
> 2years	4	88/1022	115/986	0%(0.82)	0.71 (0.40;1.28
**Year of Publication**					
<2000	2	4/418	8/392	0%(0.34)	0.60(0.12;3.00)
≥ 2000	2	84/604	107/594	0% (0.96)	0.73 (0.54; 1.00)

Supp 2. Table 11: Subgroup Analysis for doubling of SrCr in RAASi *versus* placebo

Subgroup Analysis	No. Of Trials	Pooled cumulative incidence rate		Heterogeneity I^2	Odds ratio (95% CI)
		RAASi	Placebo		
**Type of diabetes**					
Type 1 DM	2	27/259	45/229	0% (0.99)	0.51 (0.30; 0.85)*
Type 2 DM	3	283/3562	356/3546	0.% (0.40)	0.76 (0.60; 0.96)*
Both					
**Albuminuria**					
Missing	2	27/259	45/229	0% (0.35)	0.51 (0.30; 0.85)*
Macroalbuminuria	2	260/1330	333/1331	0% (0.99)	0.73 (0.59; 0.89)*
Normoalbuminuria	1	23/2232	23/2215	NA	0.99 (0.56; 1.77)
**CKD status**					
CKD any stage	4	308/3769	399/3748	19% (0.30)	0.71 (0.55; 0.93)*

No CKD	1	2/52	2/27	NA	0.50 (0.07;3.76)
**HTN status**					
Yes	5	310/3821	401/3775	0% (0.43)	0.71 (0.55;0.91)*
No	0				
**DM duration**					
<10 years	1	23/2232	23/2215	NA	0.99 (0.56; 1.77)
≥ 10 years	2	27/259	45/229	0%(0.99)	0.51 (0.30;0.85)*
Missing	2	260/1330	333/1331	0% (0.99)	0.73 (0.59; 0.89)*
**Age categories**					
< 60 years	4	148/3070	203/3013	0% (0.41)	0.78 (0.62; 0.99)
≥ 60 years	1	162/751	198/762	NA	0.78 (0.62; 0.99)
**BMI categories**					
<30	1	162/751	198/762	NA	0.78(0.62; 0.99)
≥ 30	2	121/2811	158/2784	36% (0.21)	0.75 (0.50; 1.11)
Missing	2	27/259	45/229	0%(0.99)	0.51 (0.30;0.85)*
**Study size**					
< 100	1	2/52	2/27	NA	0.50 (0.07;3.76)
≥ 100	4	308/3769	399/3748	19% (0.30)	0.71 (0.55; 0.93)*
**Study duration**					
≤ 2years	1	2/52	2/27	NA	0.50 (0.07;3.76)
> 2years	4	308/3769	399/3748	19% (0.30)	0.71 (0.55; 0.93)*
**Year of Publication**					
< 2000	1	25/207	43/202	NA	0.51 (0.30;0.85)*
≥ 2000	4	285/3614	358/3573	0% (0.57)	0.75 (0.59;0.96)

Supp 2. Table 12: Subgroup Analysis for doubling of SrCr in RAASi *versus* active treatments

Subgroup Analysis	No. Of Trials	Pooled cumulative incidence rate		Heterogeneity I^2	Odds ratio (95% CI)
		RAASi	Other Anti-HTN		
**Type of diabetes**					
Type 1 DM	0				
Type 2 DM	2	99/597	151/601	0% (0.39)	0.54 (0.26; 1.12)
**Albuminuria**					
Macroalbuminuria	2	99/597	151/601	0% (0.39)	0.54 (0.26; 1.12)
Mixed	0				
**CKD status**					
CKD any stage	2	99/597	151/601	0% (0.39)	0.54 (0.26; 1.12)
No CKD	0				

**HTN status**					
Yes	2	99/597	151/601	0% (0.39)	0.54 (0.26; 1.12)
No	0				
**DM duration**					
< 10 years	1	98/579	144/567	NA	0.60 (0.45; 0.80)*
≥ 10 years	1	1/18	7/34	NA	0.23 (0.03; 2.01)
**Age categories**					
< 60 years	1	98/579	144/567	NA	0.60 (0.45; 0.80)*
≥ 60 years	1	1/18	7/34	NA	0.23 (0.03; 2.01)
**BMI categories**					
< 30	0				
≥ 30	2	99/597	151/601	0% (0.39)	0.54 (0.26; 1.12)
**Study size**					
< 100	1	1/18	7/34	NA	0.23 (0.03; 2.01)
≥ 100	1	98/579	144/567	NA	0.60 (0.45; 0.80)*
**Study duration**					
≤ 2years	0				
> 2years	2	99/597	151/601	0% (0.39)	0.54 (0.26; 1.12)
**Year of Publication**					
< 2000	1	1/18	7/34	NA	0.23 (0.03; 2.01)
≥ 2000	1	98/579	144/567	NA	0.60 (0.45; 0.80)*

Supp 2. Table 13: Subgroup Analysis for regression of albuminuria in RAASi *versus* placebo

Subgroup Analysis According to :	No. Of Trials	RAASi	Placebo	Heterogeneity I^2	Odds ratio (95% CI)
**Type of diabetes**					
Type 1 DM	3	14/266	3/175	0%(0.96)	5.81 (1.59;21.16)*
Type 2 DM	5	110/561	54/574	79%(0.01)	2.26 (0.44; 11.45)
**Albuminuria**					
Microalbuminuria	6	116/510	50/527	72%(<.001)	3.79 (1.02; 14.15)*
Normoalbuminuria	1	0/190	0/95	NA	NA
Mixed	1	8/127	7/127	NA	1.15(0.41; 3.28)
**CKD status**					
CKD any stage	1	1/23	3/27	NA	0.36 (0.04; 3.76)
No CKD	7	123/804	54/722	71% (0.01)	3.83 (1.35; 10.91)*

**HTN status**					
Yes	2	74/321	49/328	0% (0.36)	1.74 (1.02; 2.94)*
No	5	15/338	6/247	52% (0.13)	2.59 (0.42; 15.84)
Mixed	1	35/168	2/174	NA	22.63 (5.35; 95.79)*
**DM duration**					
< 10 years	5	110/561	54/574	79%(<0.01)	2.26(0.44; 11.45)
≥ 10 years	3	14/266	3/175	0% (<0.01)	5.81 (1.59; 21.16)*
**Age categories**					
< 60 years	6	81/532	48/448	35%(0.20)	2.35 (0.70; 7.93)
≥ 60 years	2	43/295	9/301	91%(<0.01)	4.88(0.29; 91.95)
**BMI categories**					
< 30	5	57/561	12/476	73%(0.01)	5.03 (1.39; 18.15)*
≥ 30	1	66/194	42/201	NA	1.95 (1.24; 307)*
Missing	2	1/72	3/72	NA	0.36 (0.04;3.76)
**Study size**					
< 100	3	6/104	4/106	66%	1.53 (0.11; 22.2)
≥ 100	5	118/723	53/643	77% (<0.01)	3.69 (1.04; 13.06)*
**Study duration**					
≤ 2years	3	110/406	46/421	82%(<0.01)	5.48 (1.35; 22.2)*
> 2years	5	14/421	11/328	35% (0.21)	1.35 (0.29; 6.31)
**Year of Publication**					
< 2000	2	5/81	1/79	NA	6.11 (0.67; 55.1)
≥ 2000	6	119/746	56/670	74% (<0.01)	2.70 (0.72; 10.13)

Supp 2. Table 14: Subgroup Analysis for regression of albuminuria in RAASi *versus* active treatment

Subgroup Analysis According to :	No. Of Trials	RAASi	Other Anti-HTN	Heterogeneity I^2	Odds ratio (95% CI)
**Type of diabetes**					
Type 1 DM	1	5/32	0/26	NA	10.6(0.56; 201.27)
Type 2 DM	9	297/1036	235/1002	31% (0.17)	1.38 (0.96; 2.00)
**Albuminuria**					
Microalbuminuria	8	248/692	194/633	0%(0.52)	1.50 (0.90; 2.52)
Mixed	2	54/376	41/395	86%(<0.01)	1.21 (0.36; 4.15)
**CKD status**					
CKD any stage	3	55/399	44/422	76% (0.01)	1.03 (0.36; 2.90)

No CKD	7	247/669	191/606	0% (0.55)	1.54 (1.01; 2.36)*
**HTN status**					
Yes	8	296/1013	232/975	32% (0.17)	1.42 (1.02; 1.97)*
No	2	6/55	3/53	68% (0.08)	1.72 (0.07; 41.25)
**DM duration**					
< 10 years	7	253/873	212/893	45%(0.09)	1.32 (0.85; 2.05)
≥ 10 years	3	49/195	23/135	0% (0.43)	2.09(0.74; 5.93)
**Age categories**					
< 60 years	4	53/248	28/245	18% (0.30)	1.76 (0.50; 6.17)
≥ 60 years	6	249/820	207/783	25%(0.25)	1.30 (0.89; 1.89)
**BMI categories**					
< 30	7	247/669	191/606	0% (0.15)	1.54(1.01; 2.36)*
Missing	3	55/399	44/422	76%(0.01)	1.03(0.36; 2.90)
**Study size**					
< 100	4	26/154	8/96	10%(0.35)	1.67 (0.43; 6.50)
≥ 100	6	276/914	227/932	49.5%(0.08)	1.40 (0.96; 2.03)
**Study duration**					
≤ 2years	7	249/911	198/872	37%(0.15)	1.37 (0.94; 1.99)
> 2years	3	53/157	37/156	37% (0.21)	1.66 (0.29; 9.43)
**Year of Publication**					
< 2000	3	52/225	25/218	0%(0.50)	2.34 (0.84; 6.54)
≥ 2000	7	250/843	210/810	22% (0.15)	1.26 (0.82; 1.93)

Supp 2. Table 15: Subgroup Analysis for all-cause mortality in RAASi *versus* placebo

Subgroup Analysis According to :	No. Of Trials	RAASi	Placebo	Heterogeneity I^2	Odds ratio (95% CI)
**Type of diabetes**					
Type 1 DM	3	10/443	16/343	0% (0.85)	0.56 (0.24; 1.33)
Type 2 DM	6	278/3980	269/3971	4% (0.39)	1.14 (0.70;1.87)
Both	2	247/4421	296/4387	60% (0.12)	0.85 (0.61; 1.19)
**Albuminuria**					
Macroalbuminuria	2	245/1330	248/1331	0% (0.49)	0.99 (0.88; 1.21)
Microalbuminuria	2	3/240	2/247	21% (0.26)	1.31 (0.13; 13.66)
Normoalbuminuria	4	82/5132	66/5025	0% (0.64)	1.25 (0.86; 1.82)
mixed	2	197/1935	251/1896	0% (0.48)	0.71 (0.42; 1.22)

**CKD status**					
CKD any stage	4	279/3769	277/3748	42% (0.16)	1.01 (0.66; 1.54)
No CKD	7	256/5075	304/4953	0% (0.51)	0.91 (0.53; 1.58)
**HTN status**					
Yes	8	530/8511	577/8463	45% (0.08)	0.96 (0.66; 1.38)
No	3	5/333	4/238	0% (0.72)	1.02 (0.25; 4.06)
**DM duration**					
< 10 years	4	33/2650	21/2640	0% (0.52)	1.46 (0.57; 3.73)
≥ 10 years	5	257/4864	312/4730	0% (0.47)	0.81 (0.57; 1.14)
Missing	2	245/1330	248/1331	0% (0.49)	0.99(0.80; 1.21)
**Age categories**					
< 60 years	8	180/6158	175/6043	0% (0.45)	1.05 (0.68; 1.62)
≥ 60 years	3	355/2686	406/2658	59% (0.09)	0.85 (0.54; 1.32)
**BMI categories**					
< 30	6	411/5586	457/5468	22% (0.27)	0.92 (0.68; 1.25)
≥ 30	3	116/3005	109/2985	51% (0.13)	1.26 (0.68; 2.32)
Missing	2	8/253	15/248	0% (0.77)	0.52 (0.22; 1.24)
**Study size**					
< 100	1	0/46	1/46	NA	0.33 (0.01; 8.22)
≥ 100	10	535/8798	580/8655	31% (0.16)	0.97 (0.70; 1.34)
**Study duration**					
≤ 2years	2	3/240	2/247	21% (0.20)	1.31 (0.13; 13.66)
> 2years	9	532/8604	579/8454	32% (0.16)	0.95 (0.71; 1.26)
**Year of Publication**					
< 2000	3	11/350	17/345	0% (0.12)	0.66 (0.25; 1.75)
≥ 2000	8	524/8844	564/8356	38% (0.12)	1.1 (0.72; 1.41)

Supp 2. Table 16: Subgroup Analysis for all-cause mortality in RAASi *versus* active treatments

		Pooled cumulative incidence rate			
**Subgroup Analysis According to :**	**No. Of Trials**	**RAASi**	**Other Anti-HTN**	**Heterogeneity I^2**	**Odds ratio (95% CI)**
**Type of diabetes**					
Type 1 DM	1	0/25	3/27	NA	0.14 (0.01; 2.80)
Type 2 DM	9	190/2089	174/2025	0% (0.91)	1.00 (0.67; 1.49)
**Albuminuria**					
Macroalbuminuria	3	88/622	91/628	23% (0.27)	0.66 (0.22; 2.03)
Microalbuminuria	3	4/477	6/477	0%(0.77)	0.67 (0.18; 2.42)

Normoalbuminuria	1	3/301	2/303	NA	1.52 (0.25; 9.13)
Mixed	3	95/714	78/644	0% (0.62)	1.08 (0.64; 1.81)
**CKD status**					
CKD any stage	5	108/936	110/914	0% (0.52)	0.80 (0.33; 1.95)
No CKD	5	82/1178	67/1138	0% (0.83)	1.10 (0.68; 1.79)
**HTN status**					
Yes	9	171/1850	158/1818	0% (0.75)	0.92 (0.50; 1.71)
No	1	19/264	19/234	NA	0.88 (0.45; 1.71)
**DM duration**					
< 10 years	6	102/1403	86/1333	0% (0.88)	1.04 (0.66; 1.65)
≥ 10 years	3	1/132	8/152	0% (0.63)	0.25 (0.04;1.52)
Missing	1	87/579	83/567	NA	1.03 (0.74; 1.43)
**Age categories**					
< 60 years	5	182/1318	164/1238	0% (0.59)	0.98 (0.47; 2.02)
≥ 60 years	5	8/796	13/814	0% (0.76)	0.74 (0.27;2.00)
**BMI categories**					
< 30	7	83/1253	70/1217	0%(0.68)	0.95 (0.41; 2.18)
≥ 30	3	107/861	107/835	0% (0.59)	0.93 (0.56;1.53)
**Study size**					
< 100	2	1/43	8/61	0%(0.63)	0.25 (0.04; 1.52)
≥ 100	8	189/2071	169/1991	0% (0.94)	1.04 (0.74;1.46)
**Study duration**					
≤ 2years	3	2/425	2/426	0% (0.36)	1.01 (0.11; 8.96)
> 2years	7	188/1689	175/1626	0% (0.72)	0.93 (0.56;1.55)
**Year of Publication**					
< 2000	3	77/468	64/444	0% (0.46)	1.03 (0.35; 3.07)
≥ 2000	7	113/1646	113/1608	0% (0.78)	0.88 (0.49; 1.58)

Supp 2. Table 17: Subgroup Analysis for disruptive cough in RAASi *versus* placebo

Outcome	number of studies	RAASi	Placebo	I^2 (*p* value)	pooled odds ratio (95%CI)
**Type of Diabetes**					
T2DM	3	39/501	13/406	33% (0.22)	1.78 (0.39; 8.06)
T1DM	2	13/112	1/82	0% (0.75)	6.47 (1.16; 36.28)*
Both					
**Albumin Status**					
Microalbuminuria	3	13/158	3/128	49% (0.14)	2.38 (0.22; 25.53)
Normoalbuminuria	1	18/190	4/95	NA	2.38 (0.78; 7.24)
Mixed	1	21/265	7/265	NA	3.17 (1.32; 7.60)*
**CKD**					
No	4	31/348	7/223	25% (0.26)	2.43 (0.48; 12.31)
Missing	1	21/265	7/265	NA	3.17 (1.32; 7.60)*
**HTN**					
Normotensives	4	43/553	13/457	20% (0.29)	2.27 (0.53; 9.61)
Hypertensives	1	9/60	1/31	NA	5.29 (0.64; 43.87)
**Diabetes duration**					
Missing	1	9/60	1/31	NA	5.29 (0.64; 43.87)
< 10 years	1	4/52	0/51	NA	9.56 (0.50; 182.1)
≥ 10 years	3	39/501	13/406	33% (0.22)	1.78 (0.39; 8.06)
**Age category**					
< 60 years	5	52/613	14/488	3% (0.39)	2.67 (0.80; 8.89)
≥ 60 years	0				
**BMI category**					
Missing	1	0/46	2/46	NA	0.19 (0.01; 4.10)
< 30	2	43/507	11/411	0% (0.48)	3.07 (1.38; 43.87)*
≥ 30	1	9/60	1/31	NA	5.29 (0.64; 43.87)
**Study size**					
< 100	2	9/106	3/77	67%(0.08)	1.24 (0.05; 27.92)
≥ 100	3	43/507	11/411	0% (0.68)	3.07 (1.38; 43.87)*
**Study duration**					
≤ 2years	3	30/371	10/342	41% (0.19)	2.03 (0.32; 12.91)
> 2years	2	22/242	4/146	0% (0.39)	3.09 (0.80; 11.94)
**Year of Publication**					
< 2000	4	34/423	10/393	25% (0.39)	2.72 (0.55; 13.58)
≥ 2000	1	18/190	4/95	NA	2.38 (0.78; 7.24)

Supp 2. Table 18: Subgroup Analysis for disruptive cough in RAASi *versus* active treatment

Outcome	number of studies	RAASi	Other Anti-HTN	I^2 (*p* value)	pooled odds ratio (95%CI)
**Type of Diabetes**					
T2DM	8	89/1352	19/1407	1% (0.42)	5.88 (2.30; 14.99)
T1DM	1	8/24	0/24	NA	25.24 (1.36; 467.87)
**Albumin Status**					
Macroalbuminuria	2	12/42	0/58	0% (0.94)	23.29 (2.89; 187.83)
Microalbuminuria	4	13/657	2/675	0% (0.80)	4.30 (1.12; 16.48)
Mixed	2	68/376	17/395	77% (0.04)	9.78 (0.61, 156.59)
Normoalbuminuria	1	4/304	0/303	NA	9.18 (0.49;171.28)
**CKD**					
No	5	17/958	2/978	0% (0.87)	4.87 (1.44; 16.51)
Yes	4	80/418	17/453	59% (0.06)	10.30 (2.41; 44.03)
**HTN**					
Normotensives	0				
Hypertensives	9	97/1376	19/1431	7% (0.38)	6.57 (2.67; 16.16)
**Diabetes duration**					
Missing	1	3/208	1/228	NA	3.32 (0.34; 32.19)
< 10 years	4	74/963	18/981	39% (0.18)	5.77 (1.27; 26.10)
≥ 10 years	4	20/205	0/222	0% (0.946)	14.72 (3.34; 64.95)
**Age category**					
< 60 years	3	56/266	17/264	0% (0.48)	4.20 (1.37; 12.86)
≥ 60 years	6	41/1110	2/1167	0% (0.56)	8.12(2.26; 29.21)
**BMI category**					
Missing	2	68/376	17/395	77%(0.04)	9.78 (0.61; 156.6)
< 30	6	25/982	2/1002	0% (0.81)	6.22 (1.94; 19.92)
≥ 30	1	4/18	0/34	NA	21.4 (1.08;423.9)
**Study size**					
< 100	3	15/250	1/286	0%(0.46)	10.04 (1.81;55.79)
≥ 100	6	82/1126	18/1145	13% (0.33)	5.82 (1.95; 17.43)
**Study duration**					
≤ 2years	6	81/1033	19/1070	8% (0.37)	5.16 (1.73; 15.39)
> 2years	3	16/343	0/361	0% (0.88)	17.02 (3.08; 93.92)
**Year of Publication**					
< 2000	3	50/394	18/429	0% (0.45)	3.94 (1.26; 12.33)
≥ 2000	6	47/982	1/1002	0% (0.57)	10.8 (2.98; 37.11)

Supp 2. Table 19: Subgroup Analysis for the need of additional antihypertensives in RAASi *versus* placebo

Outcome	number of studies	RAASi	Placebo	I^2 (*p* value)	pooled odds ratio (95%CI)
**Type of Diabetes**					
T2DM	5	189/445	258/451	41% (0.15)	0.32 (0.14; 0.72)*
T1DM	4	8/133	21/134	0% (0.55)	0.36 (0.13; 0.97)*
Both	1	275/2613	396/2618	NA	0.66 (0.56; 0.97)*
**Albumin Status**					
Microalbuminuria	7	97/433	161/440	31%(0.19)	0.33 (0.15; 0.71)*
Normoalbuminuria	1	1/18	4/18	NA	0.66 (0.56;0.78)*
Mixed	1	275/2613	396/2618	NA	0.40 (0.20;0.82)*
Missing	1	99/127	114/127	NA	0.21 (0.02;2.06)*
**CKD**					
No	9	470/3168	665/3176	28% (0.19)	0.42 (0.24; 0.73)*
Yes	1	2/23	10/27	NA	0.16 (0.03;0.84)*
**HTN**					
Normoalbuminuriatensives	7	14/257	52/257	0% (0.61)	0.25 (0.11; 0.58)
Hypertensives	3	458/2934	623/2946	0% (0.39)	0.60 (0.46; 0.79)
**Diabetes duration**					
< 10 years	6	192/482	269/487	39% (0.14)	0.31 (0.16;0.61)*
≥ 10 years	4	280/2709	406/2716	0% (0.72)	0.62 (0.34; 1.12)
**Age category**					
< 60 years	8	370/3027	550/3040	28% (0.09)	0.41 (0.21; 0.80)*
≥ 60 years	2	102/164	125/163	0% (0.38)	0.34 (0.16; 0.71)*
**BMI category**					
Missing	4	5/136	27/136	0% (0.52)	0.19(0.06;0.63)*
< 30	5	383/2861	535/2866	39% (0.16)	0.46 (0.26; 0.80)*
≥ 30	1	84/194	113/201	NA	0.59 (0.40; 0.89)*
**Study size**					
< 100	6	10/205	40/206	0% (0.48)	0.25 (0.10; 0.66)*
≥ 100	4	462/2986	635/2997	21% (0.13)	0.55 (0.38; 0.79)*
**Study duration**					
≤2years	2	86/240	117/247	0% (0.81)	0.59 (0.40; 0.87)*
> 2years	8	386/2951	558/2956	48% (0.81)	0.34 (0.18; 0.65)*
**Year of Publication**					
< 2000	4	8/179	24/176	19% (0.3)	0.32 (0.09; 1.23)
≥ 2000	6	464/3012	648/3027	38% (0.16)	0.45 (0.28; 0.73)*

Supp 2. Table 20: Subgroup Analysis for the need of additional antihypertensives in RAASi *versus* active treatment

Outcome	number of studies	RAASi	Placebo	I^2 (*p* value)	pooled odds ratio (95%CI)
**Type of Diabetes**					
T2DM	7	334/903	309/947	72% (<0.01)	1.48 (0.77; 2.84)
T1DM	3	17/75	17/71	0% (0.95)	1.07 (0.43; 2.68)
**Albumin Status**					
Macroalbuminuria	2	32/43	48/61	NA	1.18 (0.40; 3.52)
Microalbuminuria	4	34/190	37/189	0% (1.00)	0.90 (0.53; 1.53)
Mixed	3	91/426	52/447	86% (<l0.01)	2.57 (0.69; 9.50)
Missing	1	1/18	1/18	NA	1.00 (0.06; 17.33)
Normoalbuminuria	1	193/301	188/303	NA	1.09 (0.79; 1.52)
**CKD**					
No	5	226/486	223/483	58% (0.05)	1.04 (0.78; 1.38)
Yes	5	125/492	103/535	74% (<0.01)	1.88 (0.76; 4.61)
**HTN**					
Normotensives	7	346/905	320/947	71% (<0.01)	1.50 (0.81; 2.80)
Hypertensives	3	5/73	6/71	0% (0.99)	0.82 (0.24; 2.83)
**Diabetes duration**					
Missing	1	11/46	11/45	NA	0.97 (0.37; 2.54)
<10 years	5	286/750	243/777	80% (<0.01)	1.79 (0.74; 4.33)
≥ 10 years	4	54/182	72/196	0% (0.98)	0.96 (0.55; 1.69)
**Age category**					
< 60 years	5	96/316	58/317	65% (<0.01)	1.86 (0.78; 4.42)
≥ 60 years	5	255/662	268/701	0% (0.99)	1.05 (0.81; 1.38)
**BMI category**					
< 30	6	270/543	243/554	75% (<0.01)	1.49 (0.68; 3.27)
≥ 30	4	63/417	49/440	0% (0.75)	1.37 (0.87; 2.17)
**Study size**					
< 100	5	48/162	65/177	0% (0.99)	0.99 (53; 185)
≥ 100	5	303/816	261/841	80% (<0.01)	1.71 (0.74; 3.98)
**Study duration**					
≤2years	5	121/561	84/583	78% (<0.01)	1.71 (0.72; 4.09)
> 2years	5	230/417	242/435	0% (0.99)	1.08 (0.79; 1.48)
**Year of Publication**					
< 2000	4	108/314	82/324	79% (<0.01)	2.08 (0.67; 6.5)
≥ 2000	6	243/664	241/694	0% (1.00)	1.06 (0.81; 1.39)

Supp 2. Table 21: Meta-analysis of withdrawal reasons in RAASi *versus* placebo

Withdrawal Reason	No. Of Trials	Pooled cumulative incidence rate		Heterogeneity I^2	Odds ratio (95% CI)
		RAASi	Placebo		
**Due to hyperkalemia**	5	22/1674	8/1643	30%	1.81 (0.42; 7.76)
**Due to AKI**	3	38/1010	54/991	32%	0.69 (0.36; 1.32)
**Due to uncontrolled BP**	5	63/2321	108/2230	0%	0.50 (0.24; 1.03)
**Due to other reasons**	12	910/5028	923/4893	44%	0.98 (0.59; 1.63)

Supp 2. Table 22: Meta-analysis of withdrawal reasons in RAASi *versus* active treatments

Withdrawal Reason	No. Of Trials	Pooled cumulative incidence rate		Heterogeneity I^2	Odds ratio (95% CI)
		RAASi	Other Anti-HTN		
**Due to hyperkalemia**	1	11/579	3/567	NA	3.64 (1.01; 13.12)
**Due to AKI**	1	5/400	0/358	NA	9.97 (0.55; 180.96)
**Due to uncontrolled BP**	9	49/885	40/908	0%	1.34 (0.74; 2.42)
**Due to other reasons**	15	88/1425	106/1405	0%	0.88 (0.59; 1.33)

Supp 2. Table 23: Results for overall meta- analysis for RAASi versus Placebo or Other Anti-HTN drugs (Continuous outcomes)

Outcome	Placebo-controlled studies					
	Number of studies	Patient ratio (RAASi/placebo)	Effect size	C95% CI	I^2	*P* value *
SBP	17	6691/6501	-3.32*	-4.61; -2.03	58.0%	0.0017
DBP	17	6691/6501	-1.72*	-2.51; -0.92	74%	<0.0001
Mean BP	8	1749/1761	-1.78*	-3.05; -0.51	78%	<0.0001
SrCr	4	723/706	-13.4*	-16.78; -10.01	93%	0.9267
eGFR	12	3087/2960	-0.24	-1.45;0.98	83%	<0.0001
Albuminuria	15	3525/3390	-60.8*	-113.55; -8.06	98.30%	<0.0001

Outcome	Other Anti-HTN drugs controlled studies					
	Number of studies	Patient ratio (RAASi/Other)	Effect size	C95% CI	I²	*p* value *
SBP	17	2893/2779	0.3	-1.62; 3.00	83.0%	<0.0001
DBP	17	2893/2779	0.08	-0.8; 0.96	58%	0.0016
Mean BP	6	729/737	0.22	-1.35; 1.79	80.0%	0.0001
SrCr	8	1150/1160	0.03	-6.4; 6.10	76%	0.0001
eGFR	16	1234/1262	-0.25	-2.70; 2.21	82%	<0.0001
Albuminuria	18	1671/1712	-87.64	-182.90; 7.63	95%	<0.0001

* The effect size is the Mean difference between the end of the study and the baseline except for eGFR we used the mean difference of the annual change

**test of heterogeneity

Supp 2. Table 24A: Sensitivity analysis results for overall meta- analysis after excluding outlier studies

Outcome	Placebo-controlled studies				
	Number of studies	Effect size	95% CI	I²	*p* value *
SBP	15	-3.37	-4.45; -2.29	39.90%	0.0055
DBP	16	-1.91	-2.61; -1.21	72%	<0.0001
Mean BP	No outliers detected				
SrCr	No outliers detected				
eGFR	No outliers detected				
Albuminuria	No outliers detected				

Outcome	Other Anti-HTN drugs controlled studies				
	Number of studies	Effect size	95% CI	I²	*p* value *
SBP	15	-0.45	-2.08; 1.17	65%	0.0003
DBP	16	-0.099	-0.82; 0.62	45%	0.0252
Mean BP	No outliers detected				
SrCr	No outliers detected				
eGFR	14	0.13	-0.72; 0.98	0%	0.9701
Albuminuria	17	-30.85	-59.21; -2.50	94%	<0.0001

* The effect size is the Mean difference except for eGFR we used the annual change.

**test of heterogeneity

Supp 2. Table 24B: Sensitivity analysis results for eGFR in RMD after excluding outlier studies

Comparison	*No. of trials*	*No. of patients*	*RMD§*	*95% CI of RMD*	I²	*p* value
**RAASi vs Placebo**						
All studies reporting the outcome included	12	3087/2960	-0.82	-5.54; 3.91	86%	<0.01
Studies identified as outliers excluded*	11	2960/2806	0.55	-3.81; 4.9	83%	<0.01

**RAASi vs Other Anti-HTN**						
All studies reporting the outcome included**	16	1234/1262	-1.21	-4.51; 2.09	86%	<0.01
Studies identified as outliers excluded	14	1170/1183	-2.46	-4.36; -0.56	55%	<0.01

§ RMD: Raw mean difference
* Identified outliers (random-effects model) : "Ruggenenti 2011"
** Identified outliers (random-effects model) : "Bakris 1996", "Schnack 1996"

Supp 2. Table 24C: Sensitivity analysis results for albuminuria in SMD after excluding outlier studies

Comparison	*No. of trials*	*No. of patients*	*SMD§*	*95% CI of RMD*	I^2	*p* value
**RAASi vs Placebo**						
All studies reporting the outcome included	15	3525/3390	-1.00	-1.57; -0.44	96%	<0.0001
Studies identified as outliers excluded*	8	532/483	-0.75	-1.14; -0.37	85%	<0.01
**RAASi vs Other Anti-HTN**						
All studies reporting the outcome included**	18	1671/1712	-0.55	-0.95; -0.16	90%	<0.0001
Studies identified as outliers excluded	15	1444/1462	-0.31	-0.44; -0.18	58%	<0.01

§ SMD: standardized mean difference
*Identified outliers (random-effects model) : "Crepaldi 1998", "Bilous 2009", "Mauer 2009", "Viberti 1994", "Ahmad 1997", "Ahmad 2003", "Jerumes 2004"
** Identified outliers (random-effects model): "Fogari 1999", "Baba 2001", "Tarnow 2000"

Supp 2. Table 25: excluded studies from sensitivity analysis

Outcome	Studies excluded for RAASi vs Placebo comparison	Studies excluded for RAASi vs Other anti-HTN comparison
SBP	Ahmad 1997 ; O'hare 2000	Schnack 1996; Fuchs 2016
DBP	Ahmad 2003	Schnack1996
Mean BP	No studies excluded	No studies excluded
SrCr	No studies excluded	No studies excluded
eGFR	No studies excluded	Schnack 1996; Fogari 1997
Albuminuria	No studies excluded	Tarnow 2000
