# Supplementary material for: The effect of renin–angiotensin–aldosterone system inhibitors on continuous and binary kidney outcomes in subgroups of patients with diabetes: a meta-analysis of randomized clinical trials
Source: BMC Nephrol. 2022 Apr 28;23:161. doi: 10.1186/s12882-022-02763-1 (PMC9052620; doi:10.1186/s12882-022-02763-1)
Supplement: Supplementary file 3 — Additional file 3: Supplementary 3. [file 12882_2022_2763_MOESM3_ESM.pdf]

The Effect of Renin-Angiotensin-Aldosterone System Inhibitors on Binary and Continuous Renal Outcomes in Subgroups of Patients with Diabetes: An Extensive Meta-Analysis

Supplement 3

Table of Contents

THE EFFECT OF RENIN-ANGIOTENSIN-ALDOSTERONE SYSTEM INHIBITORS ON BINARY AND CONTINUOUS RENAL OUTCOMES IN SUBGROUPS OF PATIENTS WITH DIABETES: AN EXTENSIVE META-ANALYSIS 1

SUPP 3. FIGURE 1: ABBÉ PLOT FOR TRIALS REPORTING KIDNEY FAILURE OF RAASI *VERSUS* PLACEBO. 3

SUPP 3. FIGURE 2: ABBÉ PLOT FOR TRIALS REPORTING KIDNEY FAILURE OF RAASI *VERSUS* ACTIVE TREATMENTS 4

SUPP 3. FIGURE 3: ABBÉ PLOT FOR TRIALS REPORTING DOUBLING OF SRCR OF RAASI *VERSUS* PLACEBO 4

SUPP 3. FIGURE 4: ABBÉ PLOT FOR TRIALS REPORTING DOUBLING OF SRCR OF RAASI *VERSUS* ACTIVE TREATMENTS 5

SUPP 3. FIGURE 5: ABBÉ PLOT FOR TRIALS REPORTING REGRESSION OF ALBUMINURIA OF RAASI *VERSUS* PLACEBO 5

SUPP 3. FIGURE 6: ABBÉ PLOT FOR TRIALS REPORTING REGRESSION OF ALBUMINURIA OF RAASI *VERSUS* ACTIVE TREATMENTS 6

SUPP 3. FIGURE 7: ABBÉ PLOT FOR TRIALS REPORTING ALL-CAUSE MORTALITY OF RAASI *VERSUS* PLACEBO 6

SUPP 3. FIGURE 8: ABBÉ PLOT FOR TRIALS REPORTING ALL-CAUSE MORTALITY OF RAASI *VERSUS* ACTIVE TREATMENT 7

SUPP 3. FIGURE 9: FUNNEL PLOT FOR TRIALS REPORTING KIDNEY FAILURE OF RAASI *VERSUS* PLACEBO 7

SUPP 3. FIGURE 10: FUNNEL PLOT FOR TRIALS REPORTING KIDNEY FAILURE OF RAASI *VERSUS* ACTIVE TREATMENT 8

SUPP 3. FIGURE 11: FUNNEL PLOT FOR TRIALS REPORTING DOUBLING OF SRCR OF RAASI *VERSUS* PLACEBO 8

SUPP 3. FIGURE 12: FUNNEL PLOT FOR TRIALS REPORTING DOUBLING OF SRCR OF RAASI *VERSUS* ACTIVE TREATMENT 9

SUPP 3. FIGURE 13: FUNNEL PLOT FOR TRIALS REPORTING REGRESSION OF ALBUMINURIA OF RAASI *VERSUS* PLACEBO 9

SUPP 3. FIGURE 14: FUNNEL PLOT FOR TRIALS REPORTING REGRESSION OF ALBUMINURIA OF RAASI *VERSUS* ACTIVE TREATMENT 10

SUPP 3. FIGURE 15: FUNNEL PLOT FOR TRIALS REPORTING ALL-CAUSE MORTALITY OF RAASI *VERSUS* PLACEBO 10

Supp 3. Figure 16: Funnel plot for trials reporting all-cause mortality of RAASi *versus* active treatment 11

Supp 3. Figure 1: Abbé plot for trials reporting kidney failure of RAASi *versus* placebo.

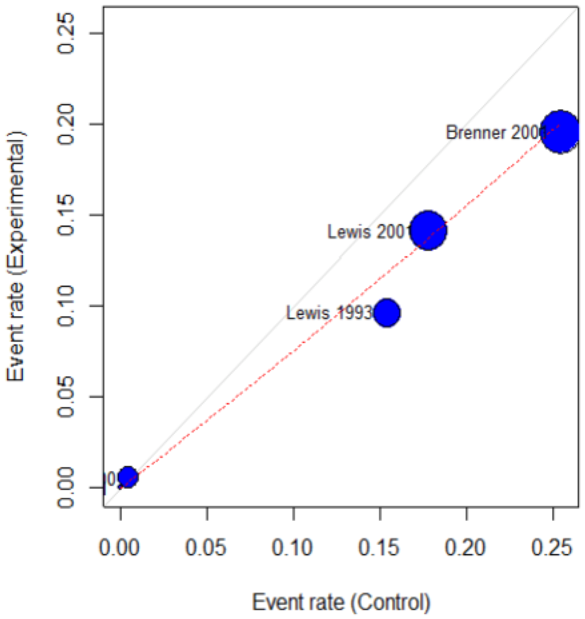

We can see that the event risks vary between studies, and e can see here that the dashed red line is in the bottom right sector of the Abbé plot. That means that the event risks in the intervention group (RAASi) are lower than the event risks in the comparator group (Placebo). This indicates a protective effect of RAASi from kidndy failure.

Supp 3. Figure 2: Abbé plot for trials reporting kidney failure of RAASi *versus* active treatments

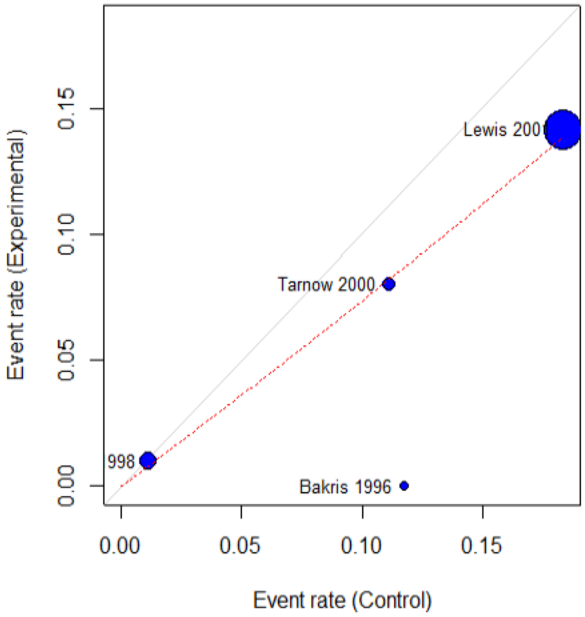

We can see that the event risks vary between studies, and all studies lie in the bottom right sector indicating a protective effect of RAASi vs Other Anti-HTN. The Bakris 1996 has very low event risks in RAASi group comparatively to the other included studies

Supp 3. Figure 3: Abbé plot for trials reporting doubling of SrCr of RAASi *versus* placebo

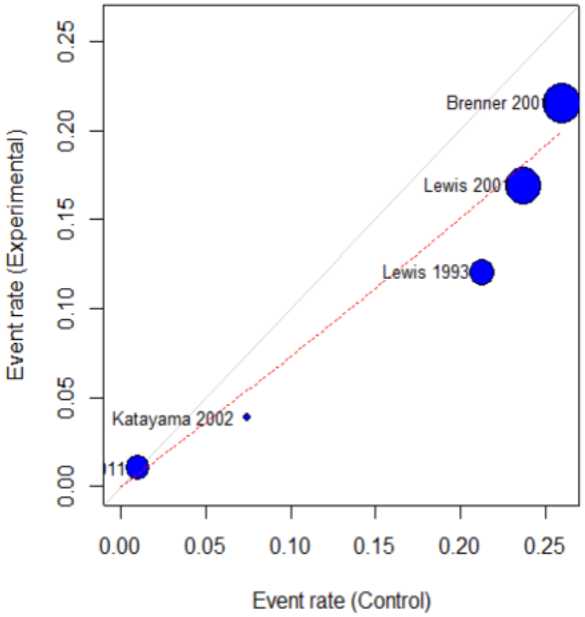

We can see that the event risks vary between studies. The event risks in RAASi arm are lower than the event risks in Placebo arm. This indicates a protective effect of RAASi from doubling of SrCr.

Supp 3. Figure 4: Abbé plot for trials reporting doubling of SrCr of RAASi *versus* active treatments

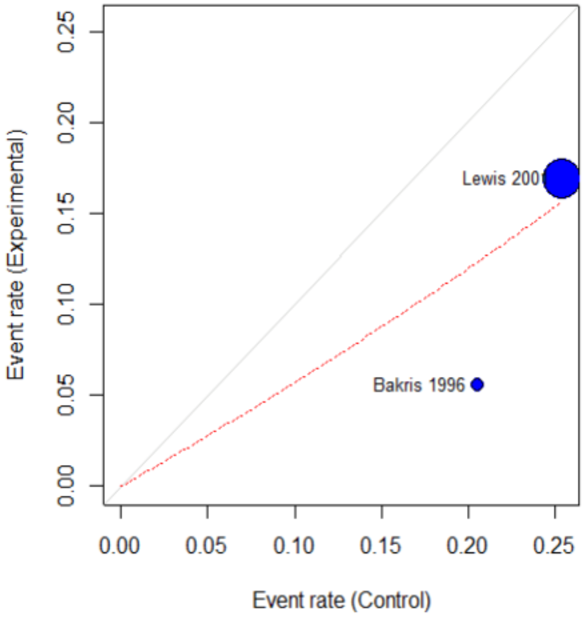

We have only two studies and both of them suggest a protective effect of RAASi vs Other Anti-HTN.

Supp 3. Figure 5: Abbé plot for trials reporting regression of albuminuria of RAASi *versus* placebo

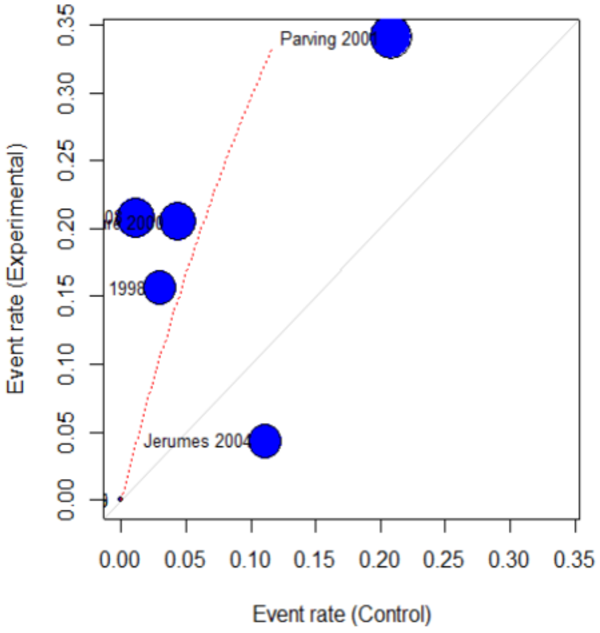

We can see that the event risks vary between studies. The dashed red line is above the diagonal grey line indicating that patients in RAASi are more likely to experiment the regression of albuminuria than patients in the Placebo group. Only one study (Jerumes) is below the grey line.

Supp 3. Figure 6: Abbé plot for trials reporting regression of albuminuria of RAASi *versus* active treatments

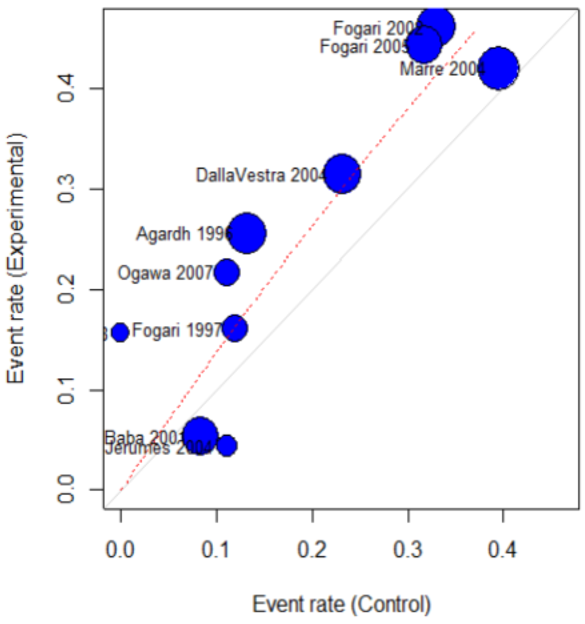

We can see that the event risks vary between studies. The dashed red line is above the diagonal grey line indicating that patients in RAASi are more likely to experiment the regression of albuminuria than patients using other Anti-HTN drugs.

Supp 3. Figure 7: Abbé plot for trials reporting all-cause mortality of RAASi *versus* placebo

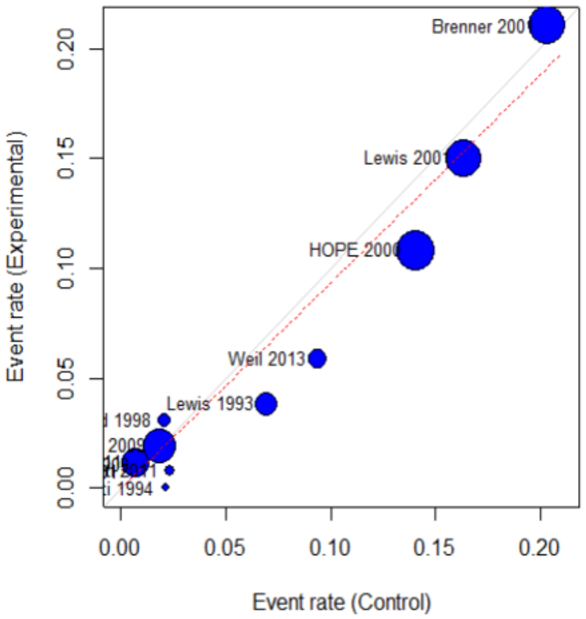

We can see that the event risks vary between studies, and that the dashed red line not far from the diagonal gray line of the Abbé plot. That means that the event risks both arms are similar.

Supp 3. Figure 8: Abbé plot for trials reporting all-cause mortality of RAASi *versus* active treatment

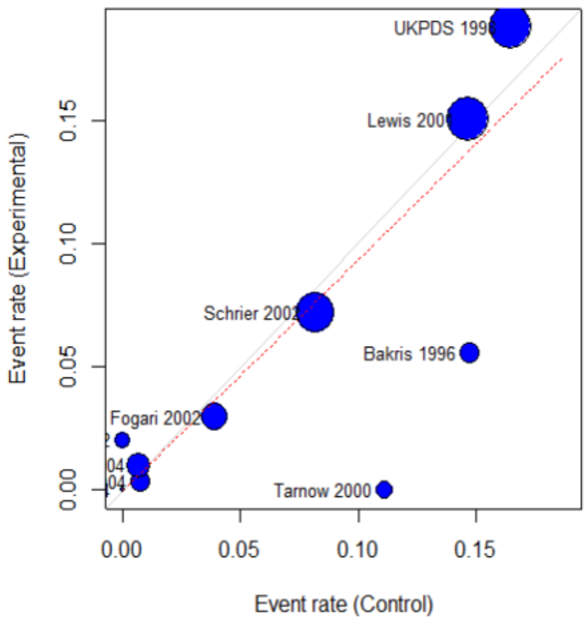

We can see that the event risks vary between studies, and that the dashed red line not far from the diagonal gray line of the Abbé plot. That means that the event risks are similar in RAASi and Other Anti-HTN arms.

Supp 3. Figure 9: Funnel plot for trials reporting kidney failure of RAASi *versus* placebo

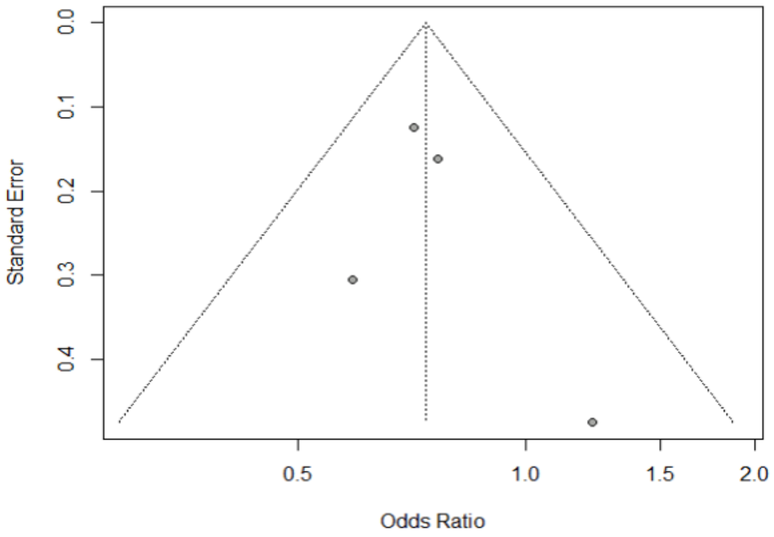

All studies lie within the stripped lines and are symmetrically around the pooled effect size. That indicates that there is no publication bias.

Supp 3. Figure 10: Funnel plot for trials reporting kidney failure of RAASi *versus* active treatment

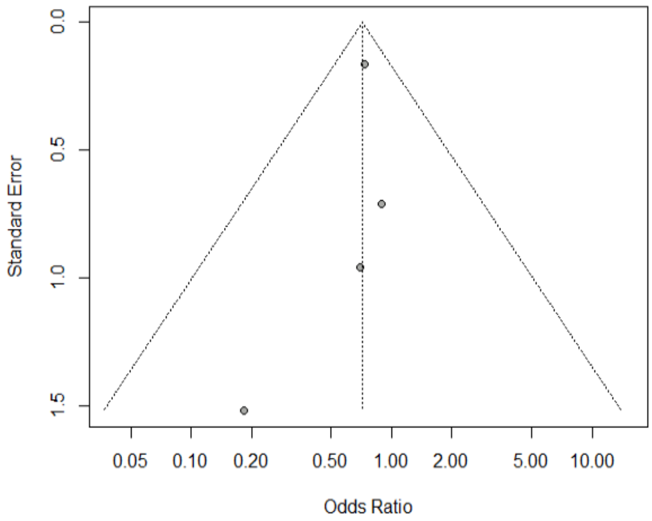

The funnel indicates no publication bias

Supp 3. Figure 11: Funnel plot for trials reporting doubling of SrCr of RAASi *versus* placebo

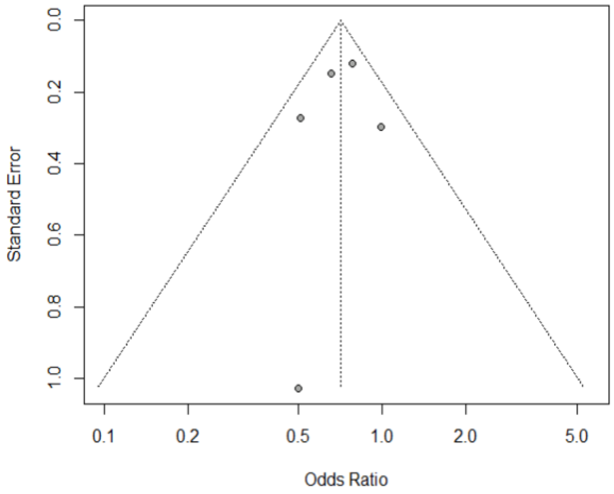

The funnel indicates no publication bias

Supp 3. Figure 12: Funnel plot for trials reporting doubling of SrCr of RAASi *versus* active treatment

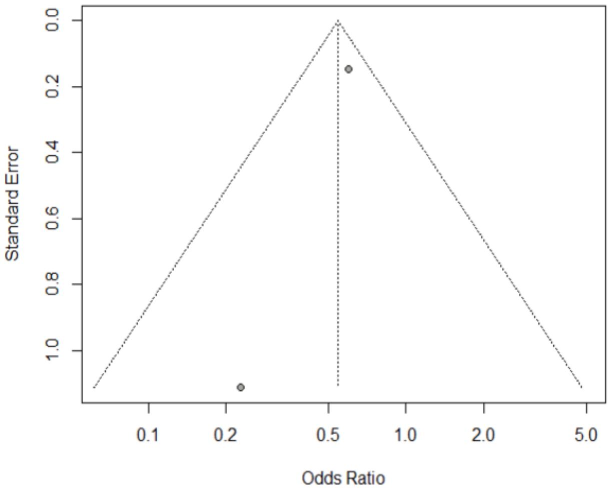

There isn’t a sufficient number of studies to make conclusions about publication bias.

Supp 3. Figure 13: Funnel plot for trials reporting regression of albuminuria of RAASi *versus* placebo

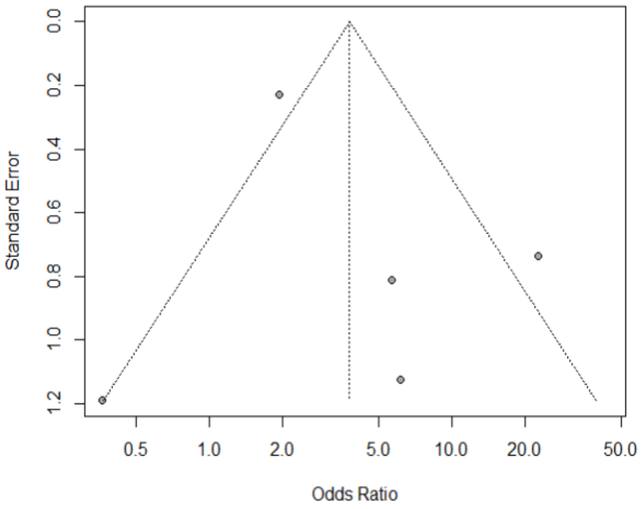

The funnel indicates the possibility of the presence of publication bias

Supp 3. Figure 14: Funnel plot for trials reporting regression of albuminuria of RAASi *versus* active treatment

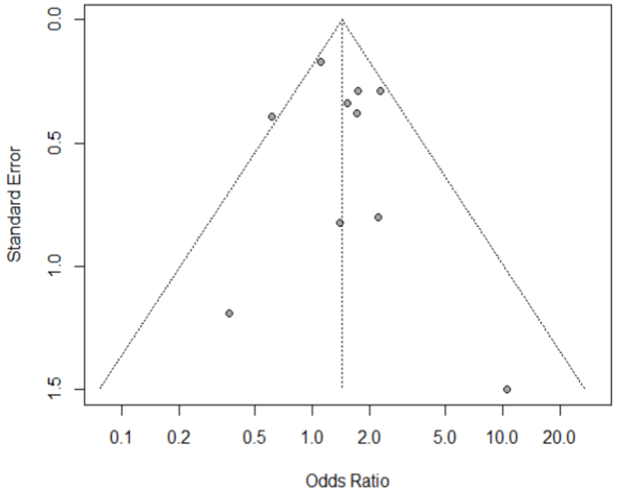

The funnel plot indicates no publication bias.

Supp 3. Figure 15: Funnel plot for trials reporting all-cause mortality of RAASi *versus* placebo

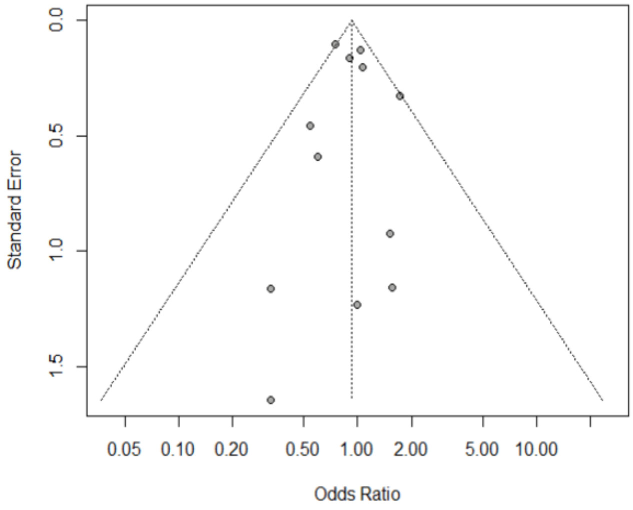

The funnel plot indicates no publication bias.

Supp 3. Figure 16: Funnel plot for trials reporting all-cause mortality of RAASi *versus* active treatment

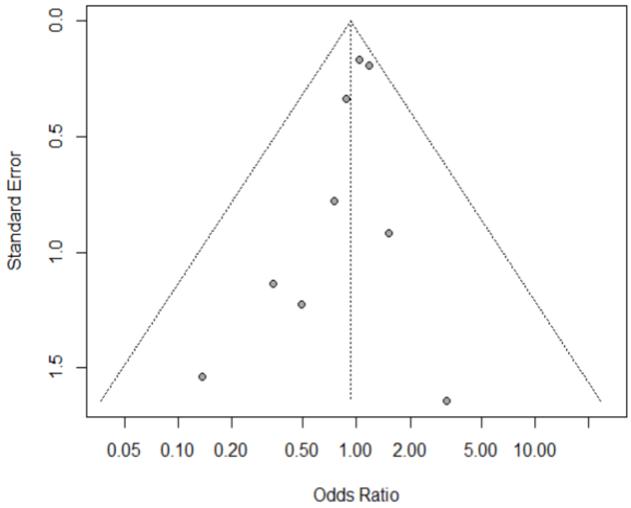

The funnel plot indicates no publication bias.
